# Patterns of Language and Visuospatial Lateralisation and Cognitive Ability in Young Children Aged 4–7 Years

**DOI:** 10.1111/desc.70193

**Published:** 2026-04-13

**Authors:** Josephine E. Quin‐Conroy, Donna M. Bayliss, Nicholas A. Badcock

**Affiliations:** ^1^ School of Psychological Science University of Western Australia Crawley Western Australia Australia

**Keywords:** children, language, lateralisation, fTCD, visuospatial

## Abstract

Previous research suggests that the development of lateralisation throughout childhood might be related to individual differences in cognitive ability, but evidence from early childhood is lacking. The current study aimed to determine if there is a relationship between patterns of language and visuospatial lateralisation and cognitive ability in young children. We assessed the cerebral lateralisation of language and visuospatial processing in a large sample of 4–7‐year‐old children (*n*  =  127) using functional transcranial Doppler ultrasound (fTCD). We found that being typically lateralised for language and visuospatial processing was related to better verbal comprehension ability for 4–5‐year‐olds. Lateralisation was not related to visuospatial ability or reading ability at any age. These findings suggest that verbal comprehension might be uniquely related to lateralisation, possibly due to its recruitment of both left and right hemispheric resources and a possible influence of mental imagery use during comprehension.

**Summary:**

- We assessed the cerebral lateralisation of language and visuospatial processing in 127 children aged 4–7 years using functional transcranial Doppler ultrasound.
- Better verbal comprehension ability was associated with being more typically lateralised for both processes in 4‐ and 5‐year‐olds.
- The crowding of cognitive processes in the same hemisphere was not associated with poorer cognitive or academic performance.

## 1 Introduction

Language and visuospatial processing are two major cognitive functions that have been extensively studied due to variations in how they lateralise in the brain. Language functions are processed more strongly in the left hemisphere of the brain than the right in approximately 85% of adults, whereas visuospatial processing activates the right hemisphere of the brain more strongly than the left in most adults (Carey and Johnstone 2014; Zago et al. 2016). Atypical lateralisation of either language or visuospatial processing (i.e. lateralisation to the atypical hemisphere or lateralisation that is, so weak that the function is considered bilateral) can occur in healthy individuals with no cognitive deficits (Flöel et al. 2001), although higher instances of atypical language lateralisation have been reported in autistic people (Lindell and Hudry 2013) and people with dyslexia (Illingworth and Bishop 2009; Papadopoulou et al. 2022). There is evidence for a relationship between language lateralisation and individual differences in language and other cognitive abilities in the general adult population—several studies have found that atypical language lateralisation is associated with poorer performance on tests of language ability and visuospatial ability (Everts et al. 2009; Mellet et al. 2014). There is evidence that visuospatial lateralisation is not related to language or visuospatial ability in adults (Powell et al. 2012), however far fewer studies have investigated visuospatial lateralisation.

As the typical pattern of left‐lateralised language and right‐lateralised visuospatial processing is the most common lateralisation pattern in adults (Badzakova‐Trajkov et al. 2010; Vingerhoets 2019), it may be that deviations from this pattern explain the link between lateralisation and cognitive ability. Levy (1969) suggested that having both functions lateralised to the same hemisphere simultaneously may be a detriment to cognitive ability as the atypical lateralisation of one function interferes with the other. This idea was further refined by subsequent research on early‐onset unilateral brain damage into the ‘functional crowding hypothesis’; whereas left‐hemispheric damage generally results in language deficits, in cases where the damage occurred in infancy, adults had relatively intact language abilities lateralised to the right hemisphere and significant deficits in spatial abilities (Lidzba et al. 2006; Satz et al. 1994; Strauss et al. 1990). These deficits were thought to reflect the crowding of both functions to the same hemisphere, affecting performance.

While the typical pattern of lateralisation is the most common in the general population, every atypical pattern can also be found in healthy individuals. Whether the impact of atypical lateralisation patterns seen in patients with unilateral brain damage extends to explaining individual differences in cognitive ability in healthy populations is less clear. A handful of studies have investigated if certain patterns of lateralisation are associated with performance on standardised cognitive measures. A review by Quin‐Conroy et al. (2023) revealed no significant differences in measures of expressive language or vocabulary knowledge in adult and adolescent participants with typical or crowded patterns (Filardi 2019; Gerrits et al. 2020; Groen et al. 2012). Powell et al. (2012) was the only study to include a measure of verbal comprehension (i.e. receptive language), and reported that crowding was related to poorer performance in verbal comprehension and perceptual organisation. Filardi (2019) investigated reading ability in adults and found that crowded participants were no worse on average than typical participants on any reading measure, but that bilateral and mixed participants (i.e., participants who were bilateral for one or both functions) outperformed the typical participants across several reading measures.

Cerebral lateralisation is generally considered to be a relatively stable trait in adults, but this is not necessarily true throughout early childhood. Early cognitive processes linked to language, such as speech and syllable discrimination, are known to be left‐lateralised in infants from birth (Bisiacchi and Cainelli 2022; Potdevin et al. 2023), and children below the age of 5 tend to show a small but significant leftward bias at the group level when listening to or producing speech (Berl et al. 2014; Kohler et al. 2015; Quin‐Conroy et al. 2026). According to a review of functional magnetic resonance imaging (fMRI) studies, language appears to be lateralised in most children by 5–7 years (Weiss‐Croft and Baldeweg 2015), although that lateralisation is weaker compared to adults, with younger children showing more right‐hemispheric activation which decreases with age (Olulade et al. 2020). There are mixed results on whether lateralisation during early development is associated with individual differences in cognitive development. Kohler et al. (2015) and Quin‐Conroy et al. (2026) both used functional transcranial Doppler ultrasound (fTCD), a non‐invasive neurophysiological technique that uses ultrasound probes to measure blood flow to the left and right hemispheres simultaneously, to estimate lateralisation in samples of 1–5‐year‐olds and 3‐year‐olds, respectively. Neither study found a significant association between degree or direction of language lateralisation and cognitive ability, before or after controlling for age. Berl et al. (2014) investigated language networks in children aged 4–12 and found that the leftward lateralisation of Wernicke's area was correlated with core language and verbal intelligence scores, but only in 4–6‐year‐olds. Taken together, this suggests that language lateralisation is established at some point within the ages of 4–7, and lateralising to the left hemisphere earlier may be related to differences in cognitive ability at that age.

Fewer studies have focused on the development of visuospatial lateralisation at young ages. Quin‐Conroy et al. (2026) found that their sample of 3‐year‐olds was not right‐lateralised for visuospatial processing on average when completing a child‐friendly line bisection task (noting that this sample was left‐lateralised for language on average). Approximately half of the samples were not significantly lateralised for visuospatial processing, and the remaining participants were equally likely to be left‐lateralised and right‐lateralised. Whilst the authors reported no differences in ability related to language lateralisation, they did find that being typically lateralised for visuospatial processing was related to having better language (but not spatial) ability. With an older sample of 5–11‐year‐old children, Ferrara et al. (2025) reported greater right‐hemisphere fMRI activation at the group level during line bisection. This right‐hemispheric lateralisation was not correlated with age, with participants across the entire age range showing variation from weakly left‐lateralised to relatively strongly right‐lateralised. Other samples of children and adolescents, including ages 6 –20, have found that greater rightward lateralisation for visual search tasks and visuospatial memory tasks was correlated with increased age, and that increased strength of visuospatial lateralisation was related to spatial ability after controlling for age (Everts et al. 2009; Groen et al. 2012; Lidzba et al. 2013). From this research, we can see a similar gradual lateralisation of visuospatial processing as there is for language, although it appears to occur at a later age.

Despite the changes in lateralisation of language and visuospatial functions across early childhood, very few studies have looked at patterns of lateralisation in children and how they relate to cognitive development. Groen et al. (2012) conducted an fTCD study with sixty participants aged 6–16 years. Of their total sample, 58% had the typical pattern of lateralisation, which is approximately equivalent to if not just under the prevalence of typical lateralisation in adults (Vingerhoets 2019), and most of the remaining participants had a crowded pattern. Groen et al. found no differences between crowded and typical participants in non‐verbal cognitive ability, vocabulary, or reading ability; however, the peak‐detection method used to estimate lateralisation from fTCD data likely over‐estimated the strength of lateralisation (see Petit et al. 2020 for more discussion), leading to potentially missing instances of true bilaterality. In stark contrast are the results of two early studies that used a behavioural finger‐tapping task to estimate lateralisation (Stellern et al. 1987; Stellern et al. 1986). Both papers recruited children aged 10–12 years with a combined sample of 125 participants. Forty‐five participants across both papers had a crowded pattern or a reversal of the typical pattern (i.e. right‐lateralised language and left‐lateralised visuospatial processing), and all atypical participants had poor performance in reading, spelling, and/or maths. The remaining participants had a typical pattern, and only seven of these participants also had poor academic performance. It should be noted, however, that these studies relied on behavioural measures of cerebral lateralisation and have yet to be replicated with more modern neurophysiological methods.

The youngest reported age group that has been used to investigate patterns of lateralisation are children 3 years of age. Quin‐Conroy et al. (2026) used novel estimates of ‘typicality’ (i.e. how close to typical a pattern is), crowding, and overall strength, and found no associations between these estimates and language or visuospatial performance. However, most of the participants were bilateral for at least one function, and only 11% of the participants were classified as typically lateralised. Given the general trend for lateralisation to increase in strength with age, and evidence for development of language and visuospatial lateralisation by the age of 7, it is likely that a sample of children above the age of 3 would have more representation of typical lateralisation. Moreover, the research linking age‐related increases in lateralisation with better language and visuospatial ability have, for the most part, focused on children 8 years or older (Everts et al. 2009; Groen et al. 2012; Lidzba et al. 2013). As there is evidence for substantial changes in lateralisation between the ages of 4 and 7, it is possible that being typically lateralised for language and visuospatial processing earlier within this age range is related to better cognitive ability. Despite this, no study has investigated combined patterns of language and visuospatial lateralisation in 4–5‐year‐olds. Groen et al. (2012) have recruited 6–8‐year‐olds, but their sample only included a small number of younger children and their method of fTCD data analysis possibly obfuscated instances of true bilaterality.

The aim of this study was to investigate the relationship between patterns of language and visuospatial lateralisation and individual differences in cognitive and academic outcomes in a large sample of 4–7‐year‐old children. Using fTCD as a child‐friendly neurophysiological method to estimate cerebral lateralisation, we first investigated how language and visuospatial processing were lateralised in the sample, including investigating the combined patterns of lateralisation. Second, we tested if language and visuospatial lateralisation, both individually and as a pattern, were related to language, visuospatial, reading, and maths performance. Following from Quin‐Conroy et al. (2026), we used measures of typicality, functional crowding, and overall strength of lateralisation to quantify different theoretically important aspects of lateralisation patterns. Based on evidence of the functional crowding hypothesis (e.g., Powell et al. 2012; Stellern et al. 1987), we predicted that functional crowding should be related to worse performance on the cognitive measures. Given that development of lateralisation has been linked to ability (Berl et al. 2014), we also predicted a positive relationship between lateralisation in the typical direction for both processes and cognitive performance. Due to evidence linking increases in strength of lateralisation with cognitive ability (e.g., Mellet et al. 2014), we tested if being more lateralised overall regardless of direction may be related to better cognitive performance. Finally, given the previous research showing that the relationship between lateralisation and cognitive performance can be different at certain ages and the substantial development in lateralisation that can occur between 4–7 years (e.g., Berl et al. 2014; Olulade et al. 2020), we investigated if there was an interaction between age and patterns of lateralisation, such that patterns of lateralisation are related to ability differently at different ages with our sample. It should be noted that we are not investigating the direction or causal nature of the relationship between lateralisation patterns and cognitive abilities, only if such a relationship exists.

## 2 Method

### 2.1 Participants

One‐hundred and thirty‐nine children aged 4–7‐years‐old were recruited to the study via advertisement posts on Facebook. All children spoke English as a first language and had no known neurological conditions (e.g., epilepsy). Ten children participated in the study but did not have any fTCD data because (i) They did not want to wear the fTCD headset (*n*  =  8), (ii) They ran out of time to complete the fTCD protocol (*n*  =  1), or (iii) Their data was not recorded due to a technical error (*n*  =  1). A further two children did attempt the fTCD protocol but were removed from the sample for not having the minimum number of acceptable epochs (7; see Quin‐Conroy et al. 2026) for either fTCD task. The final sample of 127 children consisted of, 26 4‐year‐olds, 31 5‐year‐olds, 37 6‐year‐olds, and 33 7‐year‐olds (M  =  5.61 years, SD  =  1.08 years). Sixty‐one participants were female and 66 were male. Using an adapted version of the Edinburgh Handedness Inventory (Bishop et al. 2014), 19 participants were classed as left‐handed, 106 were classed as right‐handed, and 2 did not complete the inventory. Twenty‐nine participants spoke a non‐English language at home. Seven participants were described by the parent/caregiver as having an autism or ADHD diagnosis or a mild/moderate speech delay; these participants were not removed from the sample to maximise variation of cognitive abilities and to maintain representativeness of a random sample. Maternal education and household income are shown in Table 1. A part of this sample has been reported in a previous paper (Quin‐Conroy et al. 2024). The study was conducted with approval by the University of Western Australia Human Research Ethics Committee (2020/ET000061).

**TABLE 1:** Count (percentage) of maternal demographic information related to socio‐economic status (*N*  =  127).

Demographic information		Total
Education level		
	Year 10, 11 or equivalent	2 (2%)
	Completed secondary education	5 (4%)
	Vocational education (i.e. TAFE) or apprenticeship	17 (13%)
	Tertiary Education‐ Bachelor's Degree	56 (44%)
	Tertiary Education‐ Master's or Postgraduate Degree	40 (31%)
	Other	1 (1%)
	Declined to answer	6 (5%)
	Total	127 (100%)

Household income		
	$19,999 or less	1 (1%)
	$20,000–$39,999	0 (0%)
	$40,000–$59,999	0 (0%)
	$60,000–$79,999	2 (2%)
	$80,000–$99,999	18 (14%)
	$100,000–$149,999	25 (20%)
	$150,000 or above	62 (49%)
	Declined to answer	19 (15%)
	Total	127 (100%)

### 2.2 Cognitive and Academic Measures

#### 2.2.1 Wechsler Preschool and Primary Scale of Intelligence, Fourth Edition (WPPSI‐IV)

The WPPSI‐IV is a measure of cognitive development for children aged 2 years 6 months–7 years 7 months (Wechsler 2014). We administered four subtests of the WPPSI‐IV in order to calculate two composite scores, namely a Verbal Comprehension Index (VCI) and a Visual Spatial Index (VSI). The VCI is calculated from the Information subtest, which involves answering broad general‐knowledge questions (e.g., “How many legs does a bird have?”) and is designed to measure the ability to learn, store, and retrieve general factual information, and the Similarities subtest, which involves describing how two objects or concepts are similar (e.g., “Dogs and cats are both…”) and is designed to measure concept formation and abstract reasoning. Together, the VCI measures verbal perception, comprehension, and expression, as well as aspects of crystallised intelligence, long‐term memory, and associative and categorical thinking (Wechsler 2014). The VCI has a split‐half reliability coefficient of *r * =  0.94 (Wechsler 2014).

The VSI is calculated from the Block Design subtest, which involves using blocks to recreate designs from a model and is designed to measure the ability to understand and synthesise abstract visual stimuli, and the Object Assembly subtest, which involves assembling puzzle pieces and is designed to measure visual‐perceptual organisation, non‐verbal reasoning, and trial‐and‐error learning. Together, the VSI measures spatial ability and visual perception, and aspects of visual‐motor coordination, the ability to understand figure‐ground and part‐whole relationships, and cognitive flexibility (Wechsler 2014). The VSI has a split‐half reliability coefficient of *r * = 0.89 (Wechsler 2014). Both indices are standardised to a mean of 100 and a standard deviation of 15. The subtests were administered to all participants, however twelve participants were older than 7 years 7 months and thus their raw scores could not be standardised to calculate a VCI or VSI.

#### 2.2.2 Wechsler Individual Achievement Test, Third Edition (WIAT‐III)

The WIAT‐III is a test of academic achievement across a wide range of academic skills, with scales that can be administered to children as young as 4 years (Wechsler 2016). Two subtests of the WIAT‐III, the early reading skills and maths problem solving subtests, were administered to all participants. Standard scores have a mean of 100 and a standard deviation of 15.

#### 2.2.3 Test of Word Reading Efficiency 2 (TOWRE‐2)

The TOWRE‐2 is a test of word‐level reading skills with norms for people aged 6 and over (Torgesen et al. 1999). The TOWRE‐2 requires participants to read aloud from two lists of words as fast as possible for 45 s. The first list consists of English words and measures sight word efficiency. The second list consists of pseudowords (non‐words that look/sound like English words, e.g., ‘wug’) and is used to measure phonemic decoding efficiency. The two subtests can also be used to generate a total word reading efficiency score. The TOWRE‐2 was only administered to participants aged 6 or 7 years. Additionally, the TOWRE‐2 was not administered to any of the first 25 participants. Standard scores for the TOWRE‐2 have a mean of 100 and a standard deviation of 15.

#### 2.2.4 Letter‐Sound Test (LeST)

The LeST is a test of grapheme‐phoneme knowledge (i.e. how written letters and letter combinations translate into sounds) normed for children aged 5–9 years (Larsen et al. 2015). The test involves reading a list of 51 frequently used English graphemes and saying out loud what sound each letter or group of letters make. Scores for the LeST are standardised as z‐scores, and therefore have a mean of 0 and a standard deviation of 1. The LeST was administered to all participants, but could not be standardised to z‐scores for the 4‐year‐old participants.

### 2.3 Apparatus

Cerebral blood flow velocity through the left and right middle cerebral arteries was measured simultaneously using a DWL Doppler‐Box device (DWL Elektronische Systeme, Sigen, Germany). A Diamon headset was used to secure two 2 MHz handheld transducer probes over the left and right temporal bone windows while participants completed the two fTCD computer tasks. The fTCD tasks were presented on a 59.9 cm Viewsonic vx2458‐p‐mhd monitor using PsychoPy3 (Peirce et al. 2019); participants were seated approximately 50 cm from the monitor. Event markers were sent as serial port pulses from the fTCD tasks to the DWL Doppler‐Box device via a Neurospec MMBT‐S Trigger Interface Box (NEUROSPEC AG, Stans, NW, and Switzerland) to mark each trial. Button responses to the Teddy Bear Picnic task were made using a Cedrus RB‐840 response pad (Cedrus Corporation, San Pedro, CA, United States).

### 2.4 Experimental Paradigms

The magic hat task was used to estimate the lateralisation of language, and the teddy bear picnic task was used to estimate lateralisation of visuospatial processing. Both tasks have been validated in adults as child‐friendly analogues to the word generation task and the landmark task (Quin‐Conroy et al. 2022). Trials were noted and removed during processing if the participant was disengaged during the trial, talking during the baseline period (or the food cutting period for the teddy bear picnic task), or if either fTCD signal was lost during the trial. The tasks had a maximum of 18 trials each but were discontinued early if the child wanted to stop or became disinterested in the task.

#### 2.4.1 Magic Hat Task

The magic hat task is a picture naming task where a ‘magic hat’ is turned into different objects on the screen, which participants are encouraged to name. The task includes 18 trials. Each trial begins with a 19 s normalisation period, which involves a cartoon face with a top hat moving up and down the screen, the top hat falling to the bottom of the screen, and the cartoon face waving a magic wand. At the end of the normalisation period, a voice (Australian accent, female) says “Look!”. A 16 s picture naming period follows the normalisation period, during which the hat is replaced consecutively by four random objects for 4 s each. The same voice asks what the object is (e.g., “What is it?”) at the beginning of each 4 s period and labels the object after a 3 s delay. Participants were encouraged to name the objects out loud. After the picture naming period, there is a 9 s cooldown period for the remainder of the trial, where the fourth object disappears and a celebratory sound plays (e.g., audience cheers, “Yippee!”). Finally, the voice says “Shh” as the cartoon face makes a “Shh” hand gesture to encourage the participants to be quiet for the next trial.

The picture naming period included a possible 72 unique objects in a randomised order per participant. The objects were chosen from a list of Australian English words most frequently known by 30‐month‐olds (Frank et al. 2017). Images were selected from the Bank of Standardised Stimuli database (Brodeur et al. 2014), the MultiPic database (Duñabeitia et al. 2018), and the THINGS database (Hebart et al. 2019). The background image (one of thirty blurred, symmetrical landscapes/interiors) changed each trial in a randomised order per participant.

#### 2.4.2 Teddy Bear Picnic Task

The teddy bear picnic task is a line bisection task in which participants cut food up for a teddy bear picnic by pressing a button to indicate when a knife is in the middle of a piece of food. The task has 18 total trials. Each trial includes up to six unique, approximately symmetrical images of one of 18 food types (e.g., watermelon, chocolate, fairy bread), in randomised order per participant. Each trial begins with a 20 s normalisation period, where a teddy bear ‘eats’ a piece of food from the previous round and celebrates with a random celebratory sound (during the first round, two different celebratory sounds are played). Then the screen fades to black accompanied by a ‘shh’ sound and snoring sounds to encourage the participants to not talk. Following the normalisation period and before each line bisection period, there is a 5 s period where the participant is shown six unique images of the trial's food type surrounding a plate as a voice (Australian accent, female, but different to the magic hat task's voice) labelling the food type (e.g., “Let's cut up some watermelon”).

During the line bisection period, which lasts for at least 12 s, the food images are shown consecutively in the middle of the screen on a plate. For each image, a vertical knife is displayed flashing over the food for 1 s at 1.5 s intervals. The knife's position changes with every flash, such that it moves horizontally to a random position (alternating left and right of the midpoint) until it is positioned at the midpoint of the food. The knife either starts in the midpoint and does not change position, or it lands at the midpoint on the second, third, or fourth flash. Participants were instructed to press any button on the response pad once the knife was in the middle of the food. If a button was not pressed, the knife would continue flashing without changing position until the participant did press a button. Button presses made when the knife is not in the middle of the food image have no effect on the task. When a correct response is made, a chopping sound is played to reinforce the response and the food image is replaced with the next image. The line bisection period ends when a correct button response is made after 12 s had elapsed from the start of the line bisection period. The normalisation period then follows for the next trial.

### 2.5 FTCD Data Processing

The fTCD data was processed offline using R (scripts available at REMOVED FOR ANONYMITY) according to the steps in Quin‐Conroy et al. (2024). Raw fTCD data was down‐sampled from 100 Hz to 25 Hz. Extreme artifacts, defined as values above the 99.99^th^ percentile or below the 0.01^st^ percentile, were replaced by cubic spline interpolation. Heartbeat integration was performed by averaging the data between peaks which were spaced as genuine heartbeats (i.e. at least 0.48 s apart, which assumes a maximum heart rate of 125 beats/min). Epochs were segmented as −10 s−25 s relative to event markers, which marked the start of the picture naming or line bisection period of each trial for the magic hat and teddy bear picnic tasks, respectively. The data from the left and right channels were then normalised to a mean of 100. Standard deviations of the data were calculated for each participant and data points above or below 3 SDs from 100 were removed. Individual epochs were then baseline corrected according to the baseline period of −8 s−3 s for both fTCD tasks.

Laterality indices (LIs) were estimated using generalised additive models (GAMs; Thompson et al. 2023). The GAM approach involves using several predictors to model fTCD activation, specifically to determine the difference in left and right hemisphere activation during the period of interest (POI) for each epoch. The POI for both tasks was 5 s–20 s relative to the event marker. The GAM model uses the equation

(1) $$\begin{array}{ccc} y & = & \beta_{0}+\beta_{1}.s\left(t\right)+\beta_{2}.s\left(r\right)+\beta_{3}.s\left(r,\;\mathrm{epoch}\right)+\beta_{4}.POI\\ & & +\beta_{5}.hemisphere+\beta_{6}.POI*hemisphere+\varepsilon \end{array}$$

Where y is the fTCD activation, s is a smoothing term, t is time from the start of the task, and r is relative time within each epoch. The predictors are time from the start of the experiment(β_1_), relative time within each epoch (β_2_), relative time as a factor of epoch(β_3_), POI as a boxcar function(β_4_), hemisphere(β_5_), and the interaction between POI and hemisphere(β_6_). The LI is the coefficient of the POI by hemisphere interaction(β_6_), multiplied by negative one so that a positive number indicates left hemisphere lateralisation, and a negative number indicates right hemisphere lateralisation. Individuals were categorised using 95% confidence interval overlap with zero, such that LIs that were positive and did not overlap with zero were classed a left‐lateralised, LIs that were negative and did not overlap were classed as right‐lateralised, and if the confidence interval did overlap with zero they were classed as bilateral.

We also calculated a typicality score, a crowding score, and a strength score to quantify different aspects of the participants’ lateralisation patterns (see Quin‐Conroy et al. 2026). The typicality score is a 5‐point scale quantifying each lateralisation pattern depending on how close it is to the typical pattern. The typical score is calculated by first coding the category of lateralisation for each individual function (i.e. left, bilateral, or right) as 1 if lateralised in the typical direction (i.e. left‐lateralised for language, right‐lateralised for visuospatial processing), 0 if bilateral, or −1 if lateralised in the atypical direction, and then summing those two numbers. Participants with a typical pattern (i.e. left language and right visuospatial processing) would have a typicality score of 2; a mixed typical pattern would have a score of 1; a bilateral or crowded pattern would have a score of 0; a mixed atypical pattern would have a score of −1; and participants with a reversed pattern would have a typicality score of −2.

The crowding score was calculated as the product of the language LI and the visuospatial processing LI, such that a larger positive number indicates that both functions are more strongly lateralised to the same hemisphere and a larger negative number indicates that the functions are strongly lateralised to different hemispheres. The crowding score was Winsorised to minimise the effect of relatively large LIs. The strength score is the absolute value of the crowding score, and reflects the combined strength of language and visuospatial lateralisation regardless of direction.

## 3 Results

### 3.1 Single Lateralisation

Lateralisation was estimated for participants with 7 or more acceptable epochs for either fTCD task. Of the 127 participants in our sample, 121 had at least 7 acceptable epochs for the magic hat task, 115 participants had at least 7 acceptable epochs for the teddy bear picnic task (one participant's visuospatial LI was removed due to being an outlier, z‐score > 16), and 109 participants had at least 7 acceptable epochs for both fTCD tasks. The median number of epochs for the magic hat task was 15 (IQR  =  6, min  =  7, max  =  18) and the median number of epochs for the teddy bear picnic task was 14 (IQR  =  7, min  =  7, max  =  18).

**FIGURE 1:**
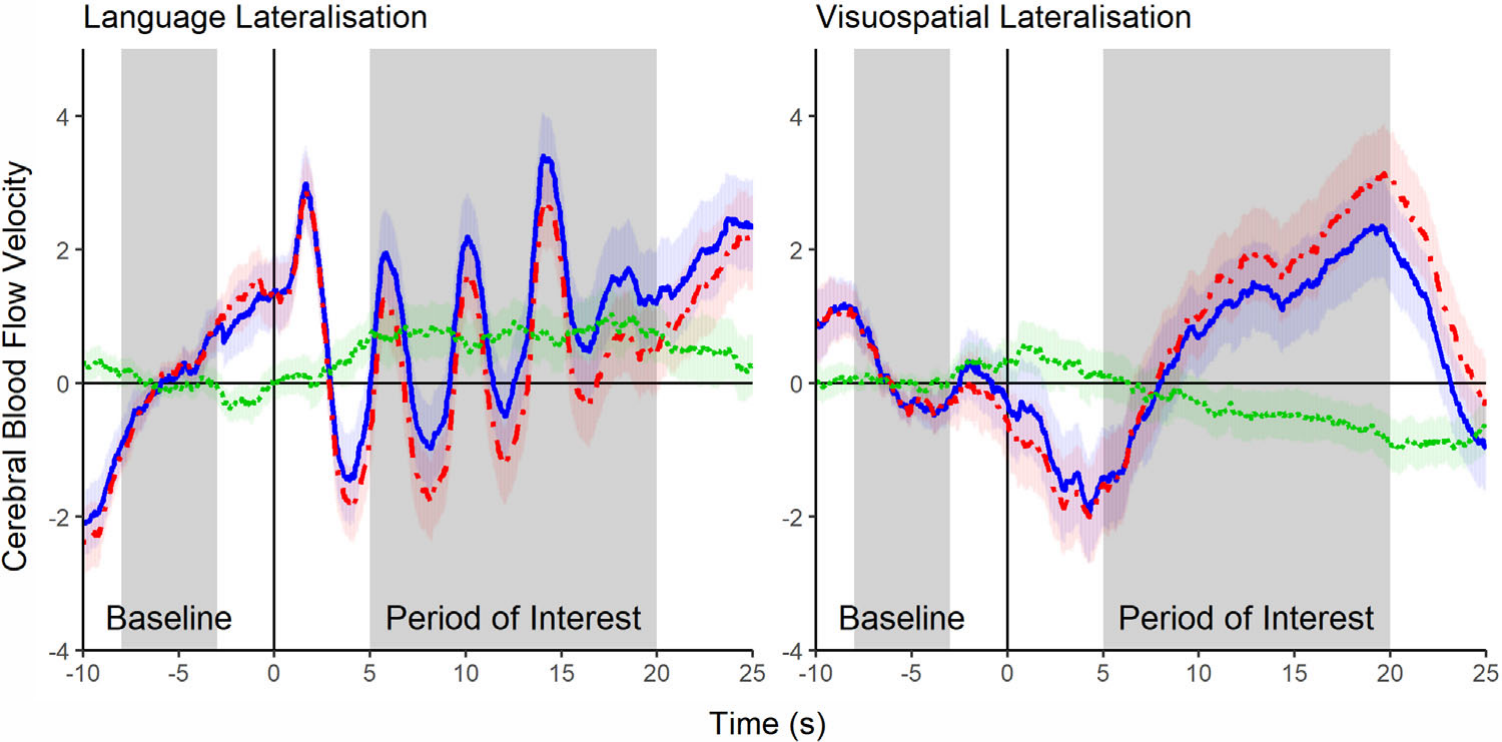
Grand‐averaged change in blood flow velocity for the language and visuospatial processing tasks. *Note*: Grand‐averaged waveforms for the magic hat task (A; n = 121) and the teddy bear picnic task (B; n = 115) showing blood flow velocity through the left (unbroken blue line) and right (dashed red line) middle cerebral arteries, and the left‐minus‐right difference (dotted green line). Shaded areas represent 95% confidence intervals.

**FIGURE 2:**
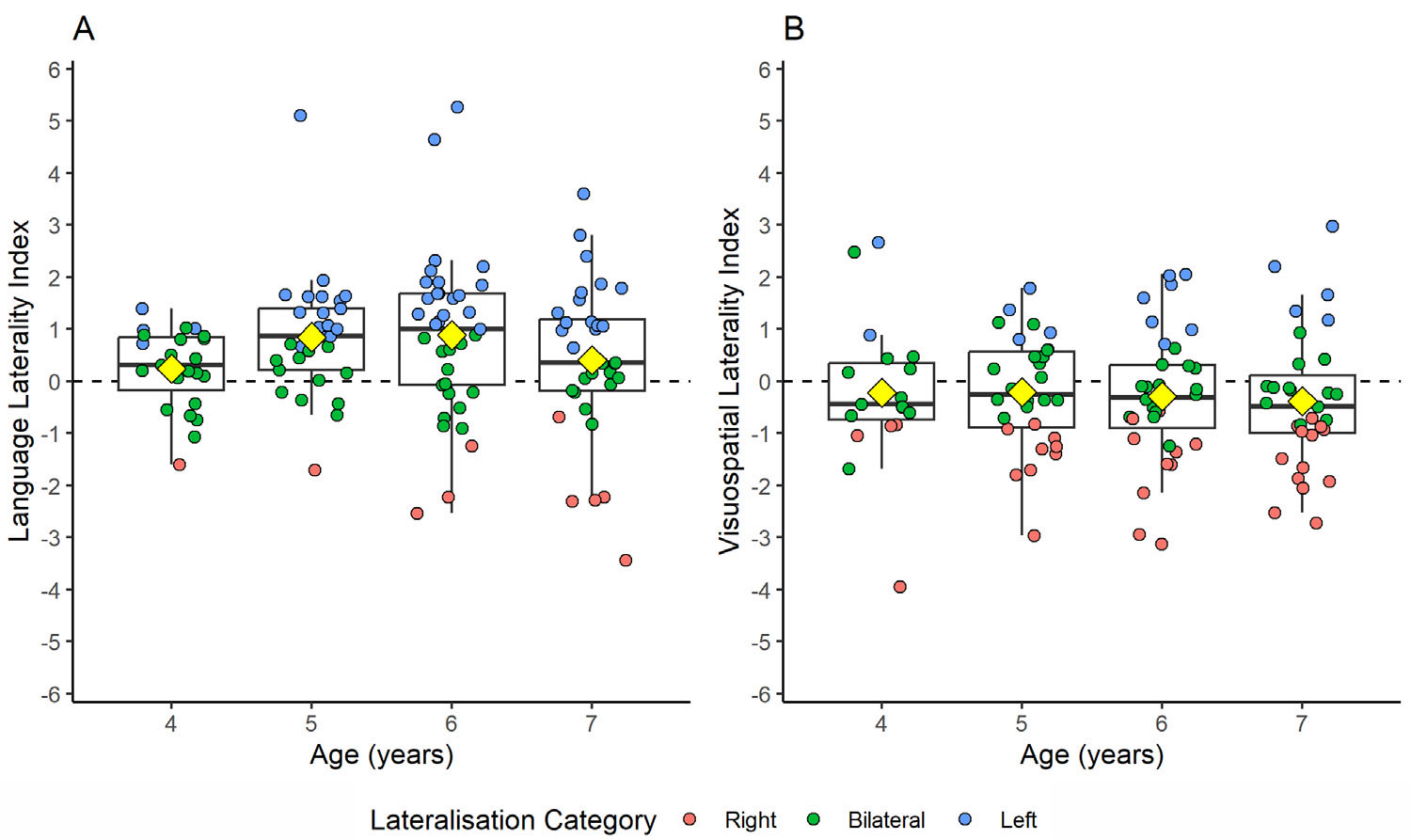
Language and visuospatial LIs by age. *Note*: Yellow diamonds represent the mean LI for each age.

The mean language LI was positive (M  =  0.62, SD  =  1.35, min  =  −3.43, max  =  5.27, skew  =  0.25, kurtosis  =  2.12) and was significantly different from zero, *t*(120)  =  5.10, *p* < 0.001, *d * =  0.46, indicating that our sample showed an overall bias to the left hemisphere for language Figures 1. A similar number of participants were categorised as left‐lateralised or bilateral for language across the total sample, but after separating participants into age groups it was evident that most of the 4‐year‐old participants were bilateral and older children were more left‐lateralised Table 2, Figure 2. Welch's t‐tests revealed that language LIs did not differ significantly between female and male participants, *t*(116.16)  =  −0.88, *p * =  0.381, *d * =  −0.16, left‐ and right‐handed participants, *t*(22.04)  =  −1.25, *p * =  0.225, *d * =  −0.32, or monolingual and multilingual participants *t*(42.47)  =  1.31, *p * =  0.196, *d * =  0.29. According to Spearman's correlations, language LIs were not significantly related to age in years, *ρ*(119)  =  0.07, *p * =  0.465, but there was a small, significant association between absolute LI and age, *ρ*(119)  =  0.19, *p * =  0.040, indicating that language LIs are stronger regardless of direction in the older children. Participants generally labelled pictures out loud when completing the task, with a low percentage of objects not being verbally labelled (Mdn  =  2.27%, IQR  =  5.77%, min  =  0%, max  =  75%, skew  =  3.69, kurtosis  =  18.08), noting that only trials where the participants were attending to the screen regardless verbal response were included as acceptable epochs. Language LIs were not correlated with the percentage of objects labelled out loud, *ρ*(119)  =  −0.17, *p * =  0.058, or with the typicality variable, *ρ*(107)  =  −0.05, *p*  =  0.613.

**TABLE 2:** Count (percentage) of lateralisation categories by age for language *(n* = 121) and visuospatial processing (*n * =  115).

		Age				
		4	5	6	7	Total
Language lateralisation	Left	4 (17%)	16 (55%)	19 (51%)	15 (47%)	54 (45%)
	Bilateral	18 (78%)	12 (41%)	15 (41%)	12 (38%)	57 (47%)
	Right	1 (4%)	1 (3%)	3 (8%)	5 (16%)	10 (8%)
	Total	23 (100%)	29 (100%)	37 (100%)	32 (100%)	121 (100%)
Visuospatial lateralisation	Left	2 (16%)	4 (13%)	7 (20%)	5 (16%)	18 (16%)
	Bilateral	13 (53%)	17 (57%)	18 (51%)	13 (42%)	61 (53%)
	Right	4 (21%)	9 (30%)	10 (29%)	13 (42%)	36 (31%)
	Total	19 (100%)	30 (100%)	35 (100%)	31 (100%)	115 (100%)

*Note*: Percentages are given within each age group and task.

Visuospatial LIs were negative on average (M  =  −0.28, SD  =  1.24, min  =  −3.94, max  =  2.98, skew  =  0.03, kurtosis  =  0.45) and significantly different from zero, *t*(114)  =  −2.45, *p * =  0.016, *d * =  −0.23, indicating a bias to the right hemisphere for visuospatial processing Figures 1. A majority of the sample were bilateral for visuospatial processing, which is true for all age groups under 7 years Table 2, Figure 2. As with language lateralisation, Welch's *t*‐tests revealed no significant differences between female and male participants, *t*(112.59)  =  0.09, *p * =  0.926, *d * =  0.02, left‐ and right‐handed participants, *t*(20.6)  =  −1.75, *p*  =  0.095, *d * =  −0.50, or monolingual and multilingual participants *t*(59.42)  =  0.28, *p * =  0.782, *d * =  −0.05. Age was not significantly correlated with visuospatial LIs, *ρ*(113)  =  −0.07, *p * =  0.465, or absolute visuospatial LIs, *ρ*(113)  =  0.08, *p * =  0.380. Visuospatial LIs were not correlated with percentage of early button responses in the teddy bear picnic task (M  =  33.95%, SD  =  21.47%, min  =  0%, max  =  91.30%), *r*(113)  =  0.09, *p * =  0.324, or with the median response time for correct button responses (Mdn  =  0.58 s, IQR  =  0.16 s, min  =  0.48 s, max  =  3.12 s), *ρ*(113)  =  0.04, *p * =  0.705. The typicality variability is also not correlated significantly with percentage of early button responses, *r*(107)  =  −0.04, *p * =  0.708, or median response time for correct button responses, *ρ*(107)  =  −0.06, *p * =  0.521.

### 3.2 Patterns of Lateralisation

**FIGURE 3:**
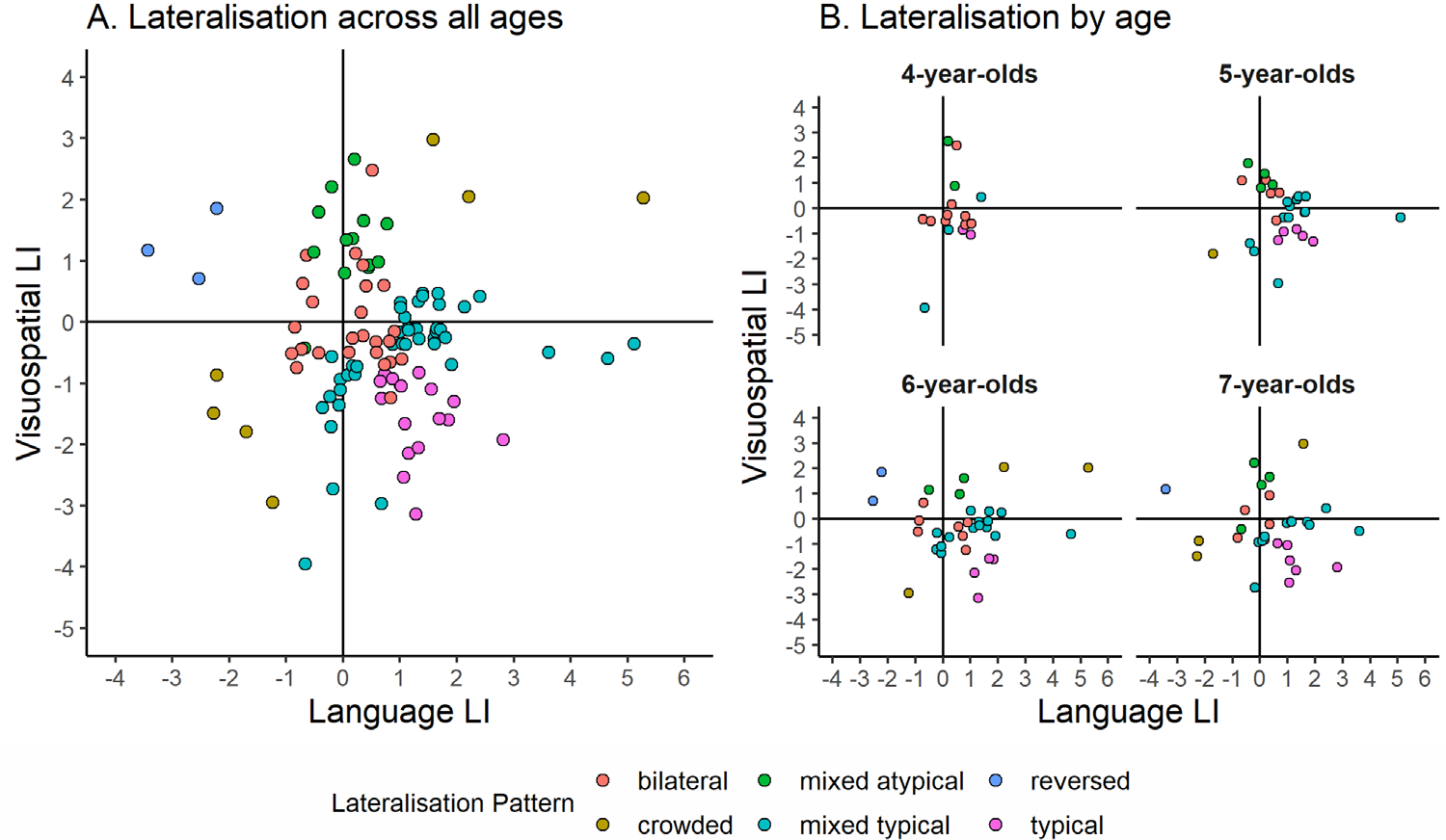
Scatterplots of LIs for language and visuospatial processing. *Note*: Yellow diamonds represent the mean LI for each age.

**FIGURE 4:**
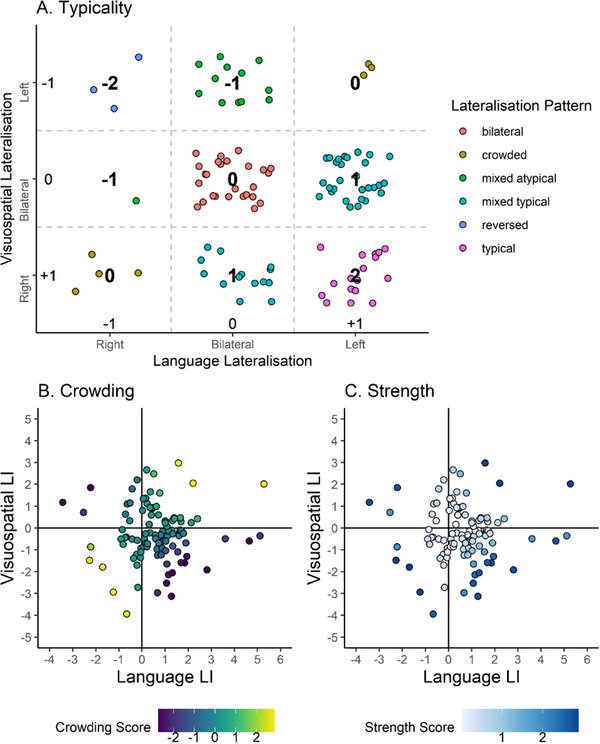
Typicality, crowding, and overall strength of lateralisation. *Note*: LI = laterality index. The typicality score (A) is a 5‐point scale quantifying how close a lateralisation pattern is to the typical pattern, calculated by coding each individual lateralisation as typical (+1), bilateral (0), or atypical (−1), and summing both numbers. The crowding score (B) is the Winsorised product of the language LI and the visuospatial processing LI, which indicates the degree of crowding to the same hemisphere or dissociation to different hemisphere. The strength score (C) is the absolute value of the crowding score, and give an indication of overall strength of lateralisation.

Table 3 shows the frequencies of each lateralisation pattern, and Figure 3 presents scatterplots of the language and visuospatial LIs. Of the 109 participants who completed both fTCD tasks, 17 participants had a typical pattern, 43 participants had a mixed typical pattern, 26 participants had a bilateral pattern, 7 participants had a crowded pattern, 13 participants had a mixed atypical pattern, and 3 participants had a reversed pattern. Language and visuospatial LIs were not significantly correlated, *r*(107)  =  0.01, *p * =  0.880. Typicality, strength, and crowding scores are colour‐coded in Figure 4.

**TABLE 3:** Frequency table of the number of participants categorised with left‐lateralised, right‐lateralised, or bilateral for language and visuospatial processing.

		Language lateralisation category			Total
		Left	Bilateral	Right	
Visuospatial Lateralisation Category	Left	3	12	3	18
	Bilateral	29	26	1	56
	Right	17	14	4	35
Total		49	52	8	109

### 3.3 Single Lateralisation and Cognitive Ability

We first tested if the lateralisation of either cognitive function alone is related to any cognitive measure using Spearman correlation analyses between age, lateralisation categorisation (coded as −1, 0, or 1 for right‐, bi‐, or left‐lateralised respectively), or lateralisation strength for language and visuospatial processing, and the language, spatial, reading, and math ability scores, presented in Table 4. Spearman correlations were used because age, lateralisation category, and strength (i.e. the absolute value of lateralisation LI) were not normally distributed. We used lateralisation categories and absolute LI as indications of direction and strength of lateralisation, respectively.

**TABLE 4:** Descriptive statistics and Spearman correlations between lateralisation, age, and ability scores.

Language Lateralisation						
Variable	*n*	M (SD)	MDN (IQR)	1. Age	2. Language category	3. Language LI strength
1. Age	121	5.64 (1.07)	6 (2)	—	—	—
2. Language category	121	0.36 (0.63)	0 (1)	0.08	—	—
3. Language LI strength	121	1.13 (0.96)	0.91 (1.15)	0.19^*^	0.48^***^	—
4. VCI	108	106.06 (14.09)	106 (18)	0.23^*^	−0.03	−0.07
5. VSI	110	101.86 (14.65)	100 (18)	−0.02	0.11	−0.06
6. LeST z‐score	97	0.29 (1.05)	0.42 (1.14)	0.27^**^	0.01	0.03
7. Early reading skills	118	101.52 (14.61)	102 (23.25)	0.08	−0.04	−0.03
8. Maths problem solving	116	102.78 (13.31)	104 (19.25)	0.07	−0.09	−0.16
9. Total word reading efficiency	56	108.5 (16.91)	107 (23)	0.05	0.11	0.05

Visuospatial lateralisation						
Variable	n	M (SD)	MDN (IQR)	1. Age	2. Visuospatial category	3. Visuospatial LI strength
1. Age	115	5.68 (1.05)	6 (2)	—	—	—
2. Visuospatial category	115	−0.16 (0.67)	0 (1)	−0.07	—	—
3. Visuospatial LI strength	115	0.98 (0.80)	0.74 (1.00)	0.08	−0.22^*^	—
4. VCI	103	106.33 (14.07)	106 (19.5)	0.20^*^	−0.19	−0.06
5. VSI	104	101.77 (14.58)	100 (18)	0.01	−0.13	−0.06
6. LeST z‐score	95	0.35 (1.01)	0.42 (1.01)	0.23^*^	0.01	−0.10
7. Early reading skills	112	102.06 (14.41)	102.5 (23)	0.04	−0.23^*^	0.08
8. Maths problem solving	110	103.33 (13.09)	105.5 (19)	0.01	−0.14	0.00
9. Total word reading efficiency	53	108.49 (16.67)	107 (26)	0.05	0.06	−0.28^*^

*Note*: * *p* < .05, **p < .01, ***p < .001.

**FIGURE 5:**
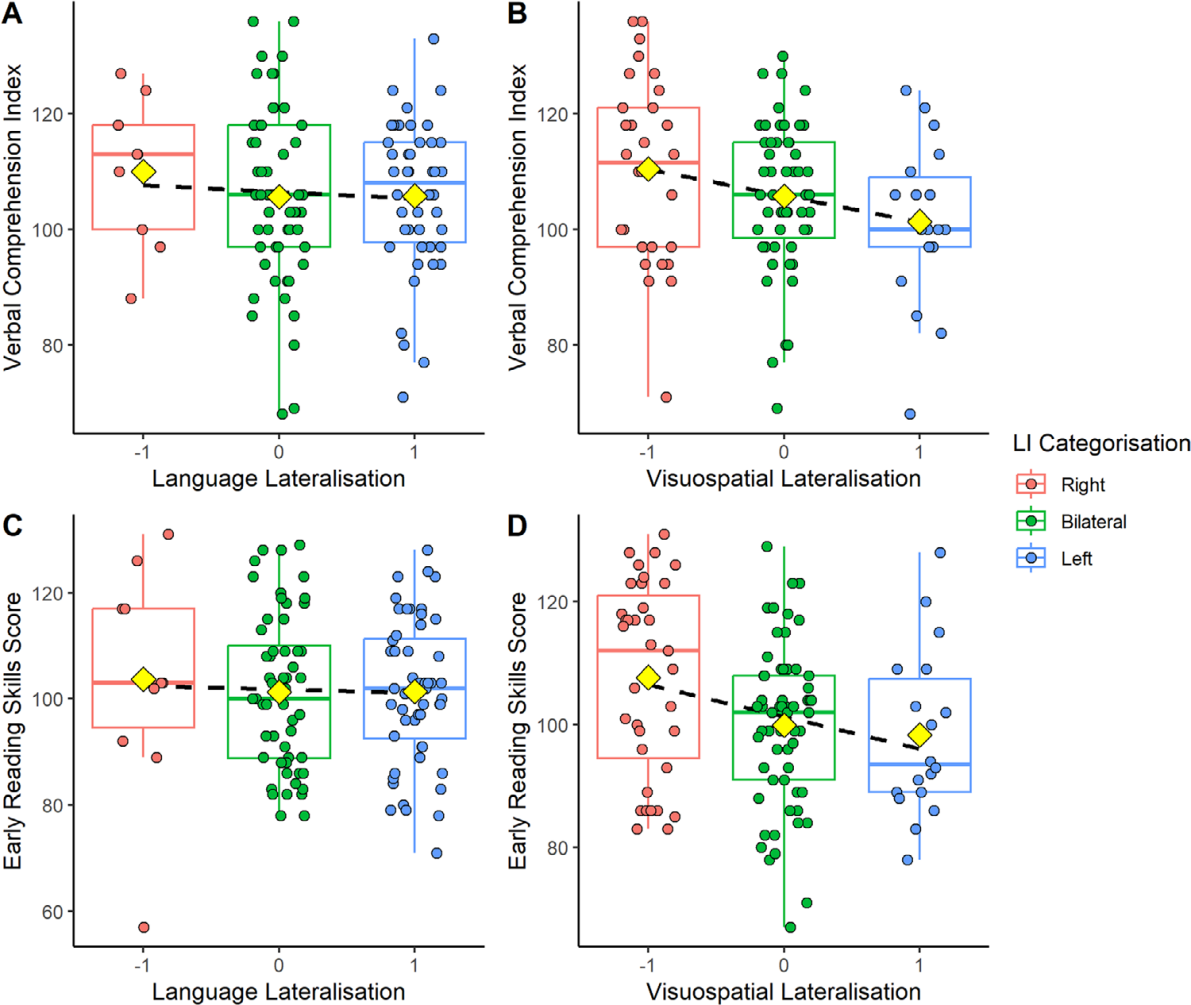
Boxplots of VCI and early reading skills scores by language and visuospatial lateralisation categories. *Note*: Yellow diamonds indicate mean VCI for each lateralisation category. Dashed black line represents the line of best fit.

Neither direction nor strength of language lateralisation was significantly correlated with any cognitive measure. Visuospatial category showed a significant, small negative correlation with early reading skills (*p * =  0.013), indicating that being right‐lateralised for visuospatial processing was related to better early reading skills Figure 5D. Strength of visuospatial lateralisation was negatively correlated with total word reading efficiency scores from the TOWRE‐2 (*p * =  0.045), indicating that being more strongly lateralised for visuospatial processing was related to poorer reading. Because age was correlated with both VCIs and LeST z‐scores, we then ran multiple regressions with lateralisation category, lateralisation strength, and age as predictors and VCI or LeST z‐scores as dependent variables to test whether lateralisation is related to either measure after controlling for age Table 5. Language lateralisation was not a significant predictor of VCI or LeST z‐scores. Visuospatial lateralisation was a significant predictor of VCI when controlling for age such that a higher VCI is associated with right‐lateralised visuospatial processing Figure 5B.

**TABLE 5:** Results of single lateralisation regression analyses.

Dependent Variable	Predictor	*b*	SE *b*	β	SE β	*t*	*p*	R^2^	Adj. R^2^	df	F	*p*
VCI	Constant	86.63	7.33			11.82	<0.001	0.08	0.06	3, 104	3.17	0.027
	Language LI strength	−1.81	1.47	−0.12	0.10	−1.23	0.222					
	Language LI category	−0.58	2.19	−0.03	0.10	−0.26	0.794					
	Age	3.91	1.32	0.30	0.10	2.95	0.004					
LeST z‐score*	Constant	−1.96	0.83			−2.37	0.020	0.08	0.05	3, 93	2.83	0.043
	Language LI strength	−0.01	0.10	−0.01	0.10	−0.10	0.924					
	Language LI category	0.08	0.15	0.05	0.09	0.53	0.599					
	Age	0.37	0.13	0.28	0.10	2.91	0.005					
VCI	Constant	89.15	7.58			11.76	<0.001	0.13	0.10	3, 99	4.85	0.003
	Visuospatial LI strength	−2.72	1.66	−0.15	0.09	−1.64	0.104					
	Visuospatial LI category	−5.01	1.97	−0.24	0.09	−2.55	0.012					
	Age	3.47	1.33	0.26	0.10	2.61	0.010					
LeST z‐score*	Constant	−1.46	0.79			−1.86	0.066	0.07	0.04	3, 91	2.18	0.096
	Visuospatial LI strength	−0.06	0.12	−0.05	0.09	−0.50	0.616					
	Visuospatial LI category	0.04	0.13	0.03	0.09	0.28	0.780					
	Age	0.31	0.12	0.25	0.10	2.54	0.013					

*Note*. *Regressions involving the LeST z‐scores did not meet the assumption of homoscedasticity, and so were performed using weighted least‐squares regressions (Rosopa et al. 2013).

### 3.4 Patterns of Lateralisation and Cognitive Ability

We performed hierarchical multiple regressions to investigate if the lateralisation pattern scores alone or the interaction between lateralisation pattern scores and age were associated with any cognitive measure. In step one of all regressions, the predictor variables were typicality score, crowding score, strength score, and age to test if lateralisation patterns are related to cognitive measures when controlling for age. In step two, the interaction term for each pattern score and age was added to the model to test if the relationship between lateralisation pattern score and the cognitive measures depended on age. Step two was not performed for the TOWRE‐2 total word reading efficiency scores because those measures were only collected for 6‐ and 7‐year‐olds, and so there are fewer age groups to differentiate and fewer participants with that measure. For the sake of brevity, only the unique contributors of each step in the regressions are shown in this section; complete statistics for all lateralisation pattern regressions are available in Appendix A. All regressions met the assumptions of normally distributed residuals, homoscedasticity, and multicollinearity (Gignac 2023).

**FIGURE 6:**
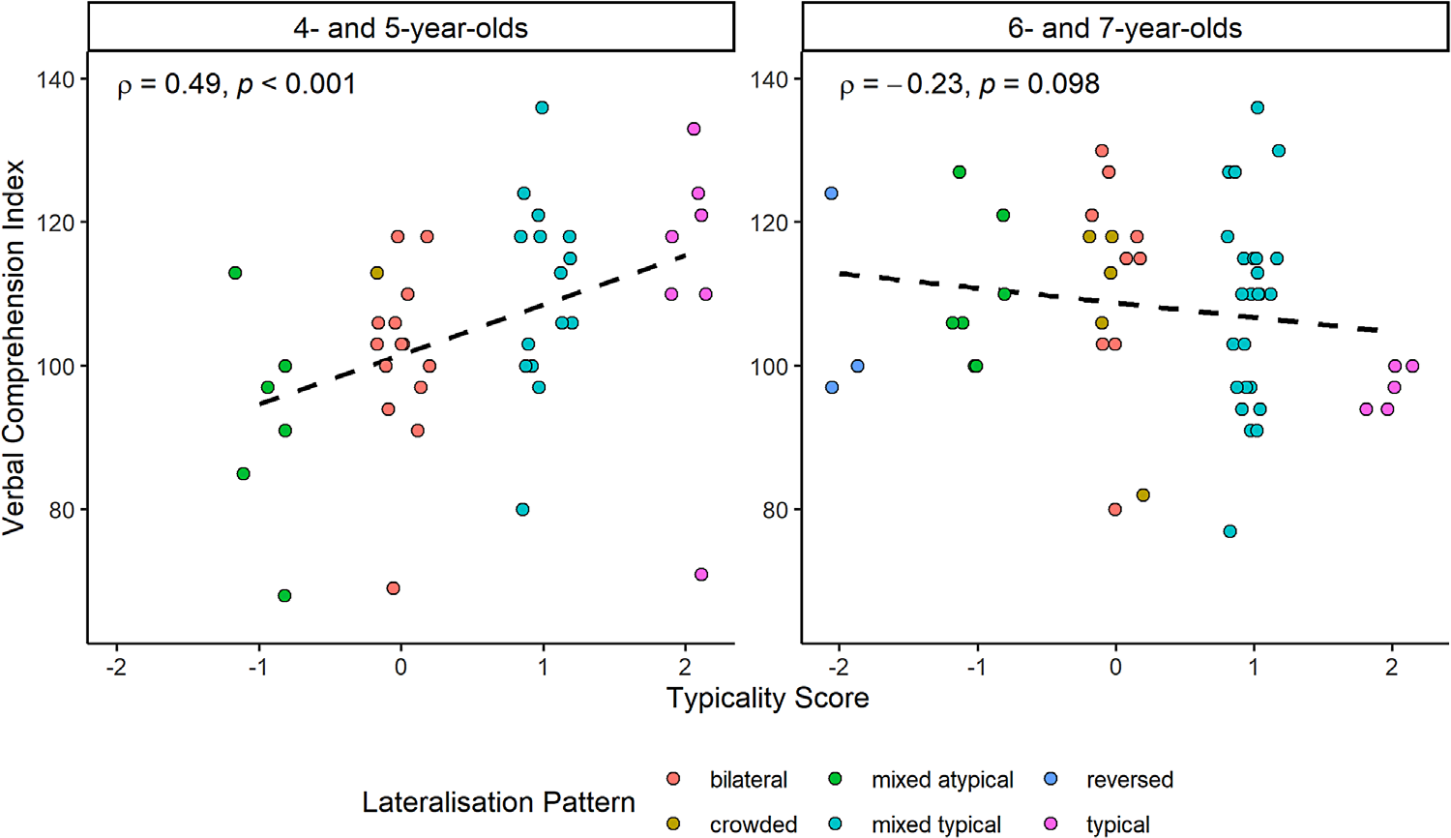
VCI and typicality scores separated by age. *Note*: Spearman's correlations are shown for each graph.

The descriptive statistics and correlations between variables are presented in Table 6, and regressions are presented in Table 7. None of the three pattern scores were significantly correlated with age. The first VCI model was not significant, indicating that pattern scores do not relate to VCI across the whole sample. The second VCI model was significant, and inspection of the individual predictors revealed that the interaction between typicality scores and age was a significant predictor of VCI. To clarify the interaction between typicality and age, we analysed the relationship using Spearman correlations between typicality and VCI for younger participants (4‐ and 5‐year‐olds) and older participants (6‐ and 7‐year‐olds; Figure 6); these groups were chosen to limit the number of *post‐hoc* analyses performed and to maximise the number of participants included in each analysis. This revealed a strong, positive relationship between VCI and typicality in the 4‐ and 5‐year‐olds, *ρ*(41)  =  0.49, *p* < 0.001, indicating that being more typically lateralised was related to better verbal comprehension in 4‐ and 5‐year‐olds. Typicality had a small, negative, non‐significant correlation with VCI in the 6‐ and 7‐year‐olds, *ρ*(52)  =  −0.23, *p * =  0.098. All other regression models revealed no significant associations between lateralisation patterns and cognitive measures at either step. Both regression models predicting LeST z‐scores were significant, however age was the only predictor that was significant in either model.

**TABLE 6:** Descriptive statistics and correlations for lateralisation pattern score, age, and cognitive measures.

Variable	*n*	M (SD)	MDN (IQR)	1. Age^1^	2. Typicality score^1^	3. Crowding score	4. Strength score^1^
1. Age^1^	109	5.72 (1.03)	6 (2)	—	—	—	—
2. Typicality score^1^	109	0.53 (0.99)	1 (1)	0.05	—	—	—
3. Crowding score	109	−0.20 (1.28)	−0.14 (0.99)	−0.07	−0.39^***^	—	—
4. Strength score^1^	109	0.89 (0.94)	0.49 (1.01)	0.05	0.16	−0.20^*^	—
5. VCI	97	106.77 (14.29)	106 (21)	0.20	0.09	0.07	−0.14
6. VSI	98	101.86 (14.87)	100 (18)	0.02	0.17	−0.10	−0.08
7. LeST z‐score	92	0.33 (1.01)	0.48 (1.03)	0.27^**^	−0.03	0.16	0.01
8. Early reading skills	106	102.57 (14.03)	103 (23.5)	0.04	0.13	0.08	0.03
9. Maths problem solving	104	103.68 (12.98)	106 (18.5)	0.01	0.00	0.12	−0.13
10. Total word reading efficiency	52	108.94 (16.50)	107 (25.5)	0.08	0.00	0.20	−0.09

*Note*: * *p* < .05, **p < .01, ***p < .001.

^1^ Spearman correlations were used for any correlation involving age, typicality scores, or strength to account for non‐normality (age, typicality scores, strength scores).

**TABLE 7:** Results of lateralisation pattern regression analyses.

Dependent Variable	Model	Predictor	*b*	SE *b*	β	SE β	*t*	*P* *(predictor)*	R^2^	Adj. R^2^	df	F	*p* (model)
VCI	1	Constant	86.49	8.36			10.35	<0.001	0.09	0.05	4, 92	2.25	0.069
		Typicality score	1.96	1.49	0.14	0.10	1.31	0.192					
		Crowding score	1.03	1.18	0.09	0.11	0.87	0.388					
		Strength score	−1.70	1.57	−0.11	0.10	−1.08	0.283					
		Age	3.74	1.57	0.27	0.11	2.55	0.012					
	2	Typicality score * Age	−4.30	1.57	−1.78	0.65	−2.73	0.008	0.17	0.10	7, 89	2.59	0.018
		Crowding score * Age	0.57	1.53	0.31	0.83	0.37	0.712					
		Strength score * Age	0.10	2.05	0.41	0.84	0.49	0.628					
VSI	1	Constant	98.23	8.76			11.21	<0.001	0.05	0.01	4, 93	1.15	0.340
		Typicality score	2.98	1.58	0.20	0.11	1.88	0.063					
		Crowding score	−0.66	1.25	−0.06	0.11	−0.53	0.601					
		Strength score	−0.71	1.67	−0.04	0.11	−0.43	0.671					
		Age	0.49	1.54	0.03	0.11	0.32	0.750					
	2	Typicality score * Age	−1.49	1.72	−0.59	0.68	−0.87	0.387	0.06	< 0.01	7, 90	0.86	0.540
		Crowding score * Age	−0.44	1.66	−0.23	0.87	−0.27	0.791					
		Strength score * Age	2.16	2.26	0.85	0.89	0.96	0.341					
LeST z‐score	1	Constant	−2.13	0.78			−2.75	0.007	0.13	0.09	4, 87	3.32	0.014
		Typicality score	0.04	0.11	0.04	0.10	0.39	0.701					
		Crowding score	0.14	0.08	0.18	0.10	1.72	0.089					
		Strength score	0.06	0.11	0.06	0.10	0.58	0.565					
		Age	0.40	0.13	0.41	0.13	3.15	0.002					
	2	Typicality score * Age	−0.09	0.14	−0.52	0.82	−0.63	0.533	0.16	0.09	7, 84	2.24	0.039
		Crowding score * Age	−0.18	0.12	−1.40	0.90	−1.56	0.123					
		Strength score * Age	−0.06	0.15	−0.36	0.89	−0.41	0.686					
Early reading skills	1	Constant	95.38	7.82			12.19	<0.001	.04	<0.01	4, 101	1.10	0.362
		Typicality score	2.37	1.45	0.17	0.10	1.63	0.106					
		Crowding score	1.54	1.13	0.15	0.10	1.36	0.177					
		Strength score	0.76	1.51	0.05	0.10	0.50	0.616					
		Age	0.97	1.36	0.07	0.10	1.72	0.476					
	2	Typicality score * Age	0.09	1.57	0.04	0.66	0.06	0.954	0.06	< 0.01	7, 98	0.93	0.490
		Crowding score * Age	−0.19	1.32	−1.11	0.73	−0.14	0.886					
		Strength score * Age	−2.44	1.77	−1.02	0.74	−1.38	0.172					
Maths problem solving	1	Constant	100.62	7.29			13.81	<0.001	0.04	<0.01	4, 99	1.00	0.410
		Typicality score	0.78	1.38	0.06	0.10	0.56	0.575					
		Crowding score	1.29	1.05	0.13	0.10	1.23	0.223					
		Strength score	−1.97	1.40	−0.14	0.10	−1.41	0.162					
		Age	0.81	1.28	0.06	0.10	0.64	0.526					
	2	Typicality score * Age	−2.62	1.45	−1.19	0.66	−1.80	0.074	0.09	0.02	7, 96	1.28	0.269
		Crowding score * Age	0.58	1.21	0.35	0.73	0.48	0.633					
		Strength score * Age	0.65	1.62	0.30	0.74	0.40	0.688					
Total word reading efficiency	1	Constant	94.83	30.26			3.13	0.003	0.06	<0.01	4, 47	0.77	0.553
		Typicality score	0.88	2.29	0.05	0.14	0.38	0.703					
		Crowding score	2.11	1.59	0.16	0.12	1.33	0.190					
		Strength score	−1.64	2.18	−0.09	0.12	−0.75	0.455					
		Age	2.53	4.72	0.16	0.29	0.54	0.594					

*Note*. For the sake of brevity, we have only included the individual predictor statistics of the interaction terms for the second models, and not the statistics for the repeated predictors (i.e. constant, typicality score, crowding score, strength score, and age). Full statistics for all models are available in Appendix A.

## 4 Discussion

The aim of the current study was to investigate the relationship between patterns of language and visuospatial lateralisation and cognitive ability in children aged 4 to 7 years. We predicted that being more typically lateralised for both language and visuospatial processing would be related to better cognitive ability. We also predicted that functional crowding would be related to poorer cognitive ability, and increased overall strength of lateralisation would be related to better cognitive ability. The first prediction was partially supported; being more typically lateralised had a strong association with better verbal comprehension scores, but only in the 4‐ and 5‐year‐olds. Contrary to our second and third predictions, crowding of language and visuospatial processing into one hemisphere and combined strength of lateralisation were not associated with cognitive ability.

Across the whole sample, language was lateralised to the left hemisphere, and visuospatial processing was lateralised to the right hemisphere. Language was bilateral in most 4‐year‐olds, but approximately half of each age group above 4 years was left‐lateralised. Additionally, strength of language lateralisation increased with age. These findings are consistent with language being lateralised in most children by the age of 5 and continuing to strengthen as children age (Olulade et al. 2020; Weiss‐Croft and Baldeweg 2015). On the other hand, visuospatial processing was only significantly right‐lateralised in approximately one‐third of our sample, and half of the participants are bilateral. Only the 7‐year‐olds had an equal representation of right‐lateralised and bilateral visuospatial processing, suggesting that the rightward lateralisation of visuospatial processing may not be established in most children until after the age of 7 years, which is consistent with previous findings that visuospatial lateralisation increases in strength throughout childhood (Everts et al. 2009; Groen et al. 2012; Lidzba et al. 2013). Ferrara et al. (2025) did not find any effects of age on visuospatial lateralisation across their sample of 5‐ to 12‐year‐old children, although they reported very few children below the age of 8 and show substantial individual variation from bilateral to right‐lateralised, similar to our findings.

The predominance of bilaterality within our results support accounts that normal development involves gradual increase in lateralisation strength throughout childhood (e.g., Everts et al. 2009). Our results also suggest that visuospatial processing follows a different developmental trajectory toward typical lateralisation than language. In our sample, and indeed in younger children aged 3 years (Quin‐Conroy et al. 2026), language is lateralised to the left hemisphere or bilateral in the majority of children, with only approximately 10% of children having significantly right‐lateralised language. This is consistent with previous research which shows that right‐hemispheric language lateralisation is rare in young children (Kohler et al. 2015). However, atypical left‐lateralised visuospatial processing does not appear to be as rare in younger children, as left‐ and right‐lateralisation was observed in an approximately equal number of 3‐ and 4‐year‐old children. Large samples of adults do show that language tends to have a much stronger bias towards the left hemisphere than visuospatial does for the right, although rightwards lateralisation of visuospatial processing is more common than atypical lateralisation (see Powell et al. 2012; Zago et al. 2016). Our data suggest that, although most children will become right‐lateralised for visuospatial processing by adulthood, at early ages they are equally likely to recruit left‐hemispheric resources as they are right‐hemispheric resources during line bisection; this may allow for more opportunity for plasticity and adaptation to environmental factors during development. Alternatively, this may point to differences in how the line bisection task is approached by young children. As was noted by Quin‐Conroy et al. (2026), younger children may be using, for example, language‐based strategies to complete the task, which would inflate the degree of left‐lateralisation (assuming the child is left‐lateralised for language). Because our data is from fTCD, we cannot localise activity within each hemisphere to determine if 3‐ and 4‐year‐olds are using different regions of the brain to complete the task; Ferrara et al. (2025) did not report regional changes in activated regions across ages for line bisection, however their study only recruited children 5 years or older. Future research could utilise fMRI, or the more child‐friendly functional near infrared spectroscopy, to determine if younger children are using different/additional brain networks when completing line bisection tasks.

Our findings suggest that being more typically lateralised for both language and visuospatial processing at 4–5 years is related to better verbal comprehension ability. This is broadly consistent with the findings of Powell et al. (2012), the only other study of this kind to also use verbal comprehension (as measured using the VCI from the Wechsler Adult Intelligence Scale third edition; WAIS‐III; Wechsler 1997) rather than language production as a measure of language ability. Powell et al. found that being typically lateralised for language and visuospatial processing was related to better verbal comprehension ability, although this was specifically in comparison to crowding which was related to poorer ability (determined using the interaction between language LIs and visuospatial LIs, which is identical to the crowding score used in the current study). Though crowding was not related to ability in our sample, Powell et al.’s sample of adults had very few bilateral, mixed atypical, or reversed participants, thus the comparison between crowding and typical participants in their paper is not dissimilar to what the typicality score demonstrates for our sample. Unlike Powell et al., we did not find that participants with typical lateralisation had better visuospatial ability than participants with atypical lateralisation.

The fact that typical lateralisation patterns are related to verbal comprehension and not visuospatial, reading, or math problem solving ability suggests that language comprehension has a unique relationship with the two hemispheres of the brain. While receptive language processes, like expressive language processes, do tend to lateralise to the left hemisphere (Bradshaw et al. 2017; Häberling et al. 2016), there is evidence that right‐hemispheric brain damage can result in minor impairments in language comprehension (Johns et al. 2008). Greater involvement of the right hemisphere has been linked to narrative comprehension during reading in children and adults (Horowitz‐Kraus et al. 2015; Horowitz‐Kraus et al. 2014), and to the processing of non‐literal and contextual information during sentence comprehension (Federmeier et al. 2008; Vigneau et al. 2011; Walenski et al. 2019). In contrast, word recognition alone tends to be left‐lateralised in adults, and children by at least the age of 7 (Deason and Marsolek 2005; Dundas et al. 2013; Kubota et al. 2019; Van der Haegen et al. 2012), and so the basic reading skills tested by our measures (i.e. sight word reading and phonological decoding) would not necessarily require both hemispheres in the same way that language or reading comprehension does. Additionally, while arithmetic functions generally have greater involvement of both hemispheres in children (Salillas et al. 2023), the early items of the math problem solving scale of the WIAT used in this study tests not only simple arithmetic (e.g., basic addition, subtraction, multiplication) but also specific math knowledge (e.g., the names of shapes, recognising numbers, how to read a clock), thus the cognitive abilities required to answer the questions vary throughout the scale. Verbal comprehension might be uniquely related to patterns of lateralisation amongst our measures due to its consistent recruitment of both left and right hemispheric resources. Greater specialisation of language and visuospatial processing to the left and right hemispheres respectively might indicate a greater ability to coordinate cognitive processes across both hemispheres, which would explain why being more typically lateralised was associated with better verbal comprehension ability in our sample. In extension, the relatively better performance of typical and mixed typical participants over mixed atypical participants would seem to indicate a degree of specialisation of each hemisphere for these processes (i.e. the left hemisphere is specialised for language, and the right hemisphere is specialised for visuospatial processing) when verbal comprehension is concerned. However, our sample had relatively few participants with mixed typical or reversed patterns; a larger sample of these atypical patterns specifically would be necessary to support claims of specialisation of each hemisphere.

A particularly interesting finding of this study is that typical lateralisation is only related to better verbal comprehension for 4‐ and 5‐year‐olds. The children aged 6 and 7 did not show a significant relationship between typical lateralisation and verbal comprehension. Quin‐Conroy et al. (2026) found no association between typicality and language ability in 3‐year‐olds, although it should be noted that Quin‐Conroy et al. reported on general language ability and not language comprehension specifically. Together, this suggests that ages 4 and 5 may be a critical period for the relationship between lateralisation and cognitive development. As most children are not typically lateralised for both functions until an older age, early lateralisation in the typical direction may be advantageous for language comprehension. Alternatively, earlier development of language comprehension abilities could trigger typical lateralisation of these cognitive functions. The fact that the relationship is not evident in older children may reflect the likely influence of time—as language ability is influenced by experience and formal education, the relationship between lateralisation and comprehension ability would become less obvious. This suggestion might explain some inconsistencies in the literature; Bishop et al. (2014) found that atypical lateralisation was more common in 4‐year‐olds with language delays, but Wilson and Bishop (2018) did not report higher instances of atypical language lateralisation in a large sample of 6–11‐year‐olds with developmental language disorder.

The relationship between language comprehension and lateralisation patterns (and particularly visuospatial lateralisation) may be clarified by understanding the involvement of mental imagery in language comprehension. Mental imagery—the ability to generate and manipulate an internal image in the ‘mind's eye’ without external visual stimuli—is thought to be an important aspect of sentence comprehension, particularly for understanding narrative or highly visual information (Bergen et al. 2007; Gambrell and Jawitz 1993; Just et al. 2004; Speed et al. 2024), and interventions focused on improving the generation of mental imagery improve listening comprehension in children (Center et al. 1999; Joffe et al. 2007). The generation of mental imagery specifically is attributed to bilateral frontoparietal networks and the left inferior temporal lobe (Liu et al. 2022; Pearson 2019), which has some overlap with the right parietal networks involved in visuospatial processing. It may be the case that being more right‐lateralised for visuospatial processing means there is less interference in homologous parietal regions in the left hemisphere that are involved in mental imagery generation. This could lead to typically lateralised individuals being better at generating mental imagery, which aids in language comprehension.

If efficiency in generating mental images could explain the relationship between lateralisation and verbal comprehension, why then is this relationship only seen at younger ages? Changes in strategies for answering questions for the verbal comprehension measurement may explain the change in relationship. In this study, verbal comprehension was measured using the Information and Similarities scales of the WPSSI‐IV. The Information scale requires participants to answer questions (e.g., “How many legs does a bird have?”), and the Similarities scale requires participants to state similarities between two objects/concepts (e.g., “Dogs and cats are both…”). Younger children may use mental imagery to answer these questions, for example by creating the mental image of a bird and counting its legs or comparing the mental images of a dog and a cat to determine their similarities. Older children (relative to the intended age range of the scales) may not need to use mental imagery to the same degree to answer these questions, as the answers could be general knowledge by that point (i.e. “Birds have two legs” and “Dogs and cats are both pets”). This might also explain why Powell et al. (2012) did find a relationship between verbal comprehension and typical lateralisation in adults; the difficulty of the verbal comprehension items in the WAIS‐III (noting that the WAIS‐III's VCI index also includes Information and Similarities scales) may mean that adults rely more on strategies like mental imagery compared to 6‐ and 7‐year‐old children completing the WPPSI‐IV. This theory would be difficult to test, because directly determining or controlling the strategies young children use to answer the verbal comprehension items may prove challenging. However, mental imagery is involved in the comprehension of narratives in children older than 5 years (Boerma et al. 2016; Gambrell and Jawitz 1993). Future studies could test if narrative comprehension ability is related to typical lateralisation in children over the age of 5 years. If a relationship is found, this would suggest that the use of mental imagery strategies may explain the relationship between typical lateralisation and verbal comprehension in 4–5‐year‐olds.

An important limitation of this research is that our data are cross‐sectional, and so any conclusions we might draw about developmental changes related to lateralisation are speculative and we cannot determine the direction of the relationship. Based on previous research, and our own findings, it appears that most young children have more bilaterally represented language in infancy which becomes further lateralised with age. However, this assumption is almost entirely based on cross‐sectional data; in Weiss‐Croft and Baldeweg's (2015) review of 39 fMRI studies that investigated language development, only one study had a longitudinal design (Szaflarski et al. 2006). Even less is known about the developmental trajectory of visuospatial lateralisation, as to our knowledge no papers have investigated this longitudinally in younger children. Given that visuospatial lateralisation in particular is associated with verbal comprehension, it seems clear that future research should investigate how language and visuospatial lateralisation develops throughout early childhood longitudinally and if individual differences in developmental trajectories are related to changes in ability, particularly in terms of the predominance of bilaterality in early childhood. Our data suggests that early shifts towards typical lateralisation are associated with improved verbal comprehension, but we would need to track the development of language and visuospatial lateralisation to confirm how children develop towards typical (or atypical) lateralisation. With a longitudinal research design, researchers could also determine if children with better verbal comprehension skills are more likely to lateralise early to the typical hemispheres, or if the timing of when children achieve typical lateralisation is associated with intraindividual improvements in ability. Depending on the scheduling of longitudinal testing, researchers could even begin to disentangle the effects of age and learning on lateralisation. For example, testing children of different ages at multiple times throughout the first school year could establish if lateralisation changes are tied to educational attainment, and if that relationship is influenced by individual differences such as language ability or early education (e.g., kindergarten or pre‐primary involvement). Another important limitation of this work is the low sample size; although our sample is large considering the age of our participants and the neurophysiological nature of our data, it is also likely to be underpowered for the regression and post‐hoc analyses that we have conducted. Our results should be regarded with caution until replicated.

In conclusion, our results show that, at the group level, language is left‐lateralised and visuospatial processing right‐lateralised in children aged 4–7 years. Better verbal comprehension ability was associated with being more typically lateralised for both processes in 4‐ and 5‐year‐olds, but this relationship was not evident in 6‐ and 7‐year‐olds. This relationship is likely influenced by visuospatial processing specifically, as more right‐lateralised visuospatial processing was related to better verbal comprehension in all ages. Together our results suggest that 4–5 years is an important period for understanding the interplay between lateralisation and cognitive development; however a lack of longitudinal data makes drawing direct conclusions about the cause of this relationship difficult. Future research should track how lateralisation changes throughout early childhood, and how these changes relate to changes in comprehension skills.

## Author Contributions

**Josephine E. Quin‐Conroy**: conceptualization, investigation, methodology, visualization, writing – original draft, writing – review and editing, formal analysis, software, project administration, data curation. **Donna M. Bayliss**: conceptualization, methodology, writing‐ review and editing, supervision. **Nicholas A. Badcock**: conceptualization, methodology, writing‐ review and editing, supervision.

## Funding

This research is supported by an Australian Government Research Training Program (RTP) Offset under Grant 10306140.

## Ethics Approval Statement

All data presented has been collected with ethics approval from the University of Western Australia Human Research Ethics Committee (2020/ET000061).

## Conflicts of Interest

The authors declare no conflicts of interest.

## AI Disclosure Statement

Generative AI tools were not used to assist this research or the development of this manuscript.

## Data Availability

Data and R scripts associated with this paper can be found and accessed on the Open Science Framework at osf.io/f56nw/.

## Appendix A Complete Statistics for Lateralisation Pattern Regressions

### A.1

Table 8 presents the coefficients, t‐values, and p‐values for all predictors in every model.

**TABLE 8:** Results of lateralisation pattern regression analyses.

Dependent Variable	Model	Predictor	*b*	SE *b*	β	SE β	*t*	*P (predictor)*	R^2^	Adj. R^2^	df	F	*p* (model)
VCI	1	Constant	86.49	8.36			10.35	<0.001	0.09	0.05	4, 92	2.25	0.069
		Typicality score	1.96	1.49	0.14	0.10	1.31	0.192					
		Crowding score	1.03	1.18	0.09	0.11	0.87	0.388					
		Strength score	−1.70	1.57	−0.11	0.10	−1.08	0.283					
		Age	3.74	1.57	0.27	0.11	2.55	0.012					
	2	Constant	82.58	11.15			7.41	<0.001	0.17	0.10	7, 89	2.59	0.018
		Typicality score	26.67	9.26	1.84	0.64	2.88	0.005					
		Crowding score	−1.94	9.06	−0.17	0.81	−0.21	0.831					
		Strength score	−8.42	12.20	−0.55	0.80	−0.69	0.492					
		Age	4.94	2.00	0.35	0.14	2.47	0.016					
		Typicality score * Age	−4.30	1.57	−1.78	0.65	−2.73	0.008					
		Crowding score * Age	0.57	1.53	0.31	0.83	0.37	0.712					
		Strength score * Age	0.10	2.05	0.41	0.84	0.49	0.628					
VSI	1	Constant	98.23	8.76			11.21	<0.001	0.05	0.01	4, 93	1.15	0.340
		Typicality score	2.98	1.58	0.20	0.11	1.88	0.063					
		Crowding score	−0.66	1.25	−0.06	0.11	−0.53	0.601					
		Strength score	−0.71	1.67	−0.04	0.11	−0.43	0.671					
		Age	0.49	1.54	0.03	0.11	0.32	0.750					
	2	Constant	103.12	12.20			8.45	<0.001	0.06	<.01	7, 90	0.86	0.540
		Typicality score	12.03	10.08	0.80	0.67	1.19	0.236					
		Crowding score	2.08	9.86	0.18	0.85	0.21	0.833					
		Strength score	−13.69	13.41	−0.86	0.85	−1.02	0.310					
		Age	−0.20	2.20	−0.01	0.15	−0.09	0.928					
		Typicality score * Age	−1.49	1.72	−0.59	0.68	−0.87	0.387					
		Crowding score * Age	−0.44	1.66	−0.23	0.87	−0.27	0.791					
		Strength score * Age	2.16	2.26	0.85	0.89	0.96	0.341					
LeST z‐score	1	Constant	−2.13	0.78			−2.75	0.007	0.13	0.09	4, 87	3.32	0.014
		Typicality score	0.04	0.11	0.04	0.10	0.39	0.701					
		Crowding score	0.14	0.08	0.18	0.10	1.72	0.089					
		Strength score	0.06	0.11	0.06	0.10	0.58	0.565					
		Age	0.40	0.13	0.41	0.13	3.15	0.002					
	2	Constant	−2.55	1.16			−2.20	0.030	0.16	0.09	7, 84	2.24	0.039
		Typicality score	0.58	0.87	0.57	0.85	0.67	0.504					
		Crowding score	1.26	0.72	1.60	0.92	1.74	0.086					
		Strength score	0.44	0.95	0.41	0.88	0.47	0.641					
		Age	0.48	0.20	0.49	0.20	2.46	0.016					
		Typicality score * Age	−0.09	0.14	−0.52	0.82	−0.63	0.533					
		Crowding score * Age	−0.18	0.12	−1.40	0.90	−1.56	0.123					
		Strength score * Age	−0.06	0.15	−0.36	0.89	−0.41	0.686					
Early reading skills	1	Constant	95.38	7.82			12.19	<0.001	0.04	<0.01	4, 101	1.10	0.362
		Typicality score	2.37	1.45	0.17	0.10	1.63	0.106					
		Crowding score	1.54	1.13	0.15	0.10	1.36	0.177					
		Strength score	0.76	1.51	0.05	0.10	0.50	0.616					
		Age	0.97	1.36	0.07	0.10	1.72	0.476					
	2	Constant	85.02	10.90			7.80	<0.001	0.06	<0.01	7, 98	0.93	0.490
		Typicality score	1.53	9.38	0.11	0.66	0.16	0.871					
		Crowding score	2.44	8.08	0.22	0.74	0.30	0.763					
		Strength score	15.44	10.88	1.03	.072	1.42	0.159					
		Age	2.75	1.92	0.20	0.14	1.43	0.156					
		Typicality score * Age	0.09	1.57	0.04	0.66	0.06	0.954					
		Crowding score * Age	−0.19	1.32	−1.11	0.73	−0.14	0.886					
		Strength score * Age	−2.44	1.77	−1.02	0.74	−1.38	0.172					
Maths problem solving	1	Constant	100.62	7.29			13.81	<0.001	0.04	<0.01	4, 99	1.00	0.410
		Typicality score	0.78	1.38	0.06	0.10	0.56	0.575					
		Crowding score	1.29	1.05	0.13	0.10	1.23	0.223					
		Strength score	−1.97	1.40	−0.14	0.10	−1.41	0.162					
		Age	0.81	1.28	0.06	0.10	0.64	0.526					
	2	Constant	96.19	10.10			9.53	<0.001	0.09	0.02	7, 96	1.28	0.269
		Typicality score	16.07	8.65	1.22	0.66	1.86	0.066					
		Crowding score	−2.21	7.39	−0.22	0.73	−0.30	0.765					
		Strength score	−6.08	9.96	−0.44	0.72	−0.61	0.543					
		Age	1.89	1.80	0.15	0.14	1.05	0.294					
		Typicality score * Age	−2.62	1.45	−1.19	0.66	−1.80	0.074					
		Crowding score * Age	0.58	1.21	0.35	0.73	0.48	0.633					
		Strength score * Age	0.65	1.62	0.30	0.74	0.40	0.688					
Total word reading efficiency	1	Constant	94.83	30.26			3.13	0.003	0.06	<0.01	4, 47	0.77	0.553
		Typicality score	0.88	2.29	0.05	0.14	0.38	0.703					
		Crowding score	2.11	1.59	0.16	0.12	1.33	0.190					
		Strength score	−1.64	2.18	−0.09	0.12	−0.75	0.455					
		Age	2.53	4.72	0.16	0.29	0.54	0.594					

## References

[desc70193-bib-0001] Badzakova‐Trajkov, G. , I. S. Häberling , R. P. Roberts , and M. C. Corballis . 2010. “Cerebral Asymmetries: Complementary and Independent Processes.” PLoS ONE 5, no. 3: e9682. 10.1371/journal.pone.0009682. 20300635 PMC2837380

[desc70193-bib-0002] Bergen, B. K. , S. Lindsay , T. Matlock , and S. Narayanan . 2007. “Spatial and Linguistic Aspects of Visual Imagery in Sentence Comprehension.” Cognitive Science 31, no. 5: 733–764. 10.1080/03640210701530748. 21635316

[desc70193-bib-0003] Berl, M. M. , J. Mayo , E. N. Parks , et al. 2014. “Regional Differences in the Developmental Trajectory of Lateralization of the Language Network.” Human Brain Mapping 35, no. 1: 270–284. 10.1002/hbm.22179. 23033058 PMC3578038

[desc70193-bib-0004] Bishop, D. V. M. , G. Holt , A. J. O. Whitehouse , and M. Groen . 2014. “No Population Bias to Left‐Hemisphere Language in 4‐year‐olds With Language Impairment.” PeerJ 2: e507. 10.7717/peerj.507. 25165624 PMC4137668

[desc70193-bib-0005] Bisiacchi, P. , and E. Cainelli . 2022. “Structural and Functional Brain Asymmetries in the Early Phases of Life: A Scoping Review.” Brain Structure and Function 227, no. 2: 479–496. 10.1007/s00429-021-02256-1. 33738578 PMC8843922

[desc70193-bib-0006] Boerma, I. E. , S. E. Mol , and J. Jolles . 2016. “Reading Pictures for Story Comprehension Requires Mental Imagery Skills [Original Research].” Frontiers in Psychology 7: 1630. 10.3389/fpsyg.2016.01630. 27822194 PMC5075566

[desc70193-bib-0007] Bradshaw, A. R. , P. A. Thompson , A. C. Wilson , D. V. M. Bishop , and Z. V. J. Woodhead . 2017. “Measuring Language Lateralisation With Different Language Tasks: A Systematic Review.” PeerJ 5: e3929. 10.7717/peerj.3929. 29085748 PMC5659218

[desc70193-bib-0008] Brodeur, M. B. , K. Guérard , and M. Bouras . 2014. “Bank of Standardized Stimuli (BOSS) Phase II: 930 New Normative Photos.” PLoS ONE 9, no. 9: e106953. 10.1371/journal.pone.0106953. 25211489 PMC4161371

[desc70193-bib-0009] Carey, D. P. , and L. T. Johnstone . 2014. “Quantifying Cerebral Asymmetries for Language in Dextrals and Adextrals With Random‐Effects Meta Analysis.” In Frontiers in Psychology, 5. 10.3389/fpsyg.2014.01128. PMC4219560 25408673

[desc70193-bib-0010] Center, Y. , L. Freeman , G. Robertson , and L. Outhred . 1999. “The Effect of Visual Imagery Training on the Reading and Listening Comprehension of Low Listening Comprehenders in Year 2.” Journal of Research in Reading 22, no. 3: 241–256. 10.1111/1467-9817.00088.

[desc70193-bib-0011] Deason, R. G. , and C. J. Marsolek . 2005. “A Critical Boundary to the Left‐Hemisphere Advantage in Visual‐Word Processing.” Brain and Language 92, no. 3: 251–261. 10.1016/j.bandl.2004.06.105. 15721958

[desc70193-bib-0012] Duñabeitia, J. A. , D. Crepaldi , A. S. Meyer , et al. 2018. “MultiPic: A Standardized Set of 750 Drawings With Norms for Six European Languages.” Quarterly Journal of Experimental Psychology 71, no. 4: 808–816. 10.1080/17470218.2017.1310261. 28326995

[desc70193-bib-0013] Dundas, E. M. , D. C. Plaut , and M. Behrmann . 2013. “The Joint Development of Hemispheric Lateralization for Words and Faces.” Journal of Experimental Psychology General 142, no. 2: 348–358. 10.1037/a0029503. 22866684 PMC4241688

[desc70193-bib-0014] Everts, R. , K. Lidzba , M. Wilke , et al. 2009. “Strengthening of Laterality of Verbal and Visuospatial Functions During Childhood and Adolescence.” Human Brain Mapping 30, no. 2: 473–483. 10.1002/hbm.20523. 18219619 PMC6871185

[desc70193-bib-0015] Federmeier, K. D. , E. W. Wlotko , and A. M. Meyer . 2008. “What's ‘Right’ in Language Comprehension: Event‐Related Potentials Reveal Right Hemisphere Language Capabilities.” Language and Linguistics Compass 2, no. 1: 1–17. 10.1111/j.1749-818X.2007.00042.x. 19777128 PMC2748422

[desc70193-bib-0016] Ferrara, K. , A. Seydell‐Greenwald , C. E. Chambers , E. L. Newport , and B. Landau . 2025. “Developmental Changes in Neural Lateralization for Visual‐Spatial Function?” Evidence From a Line‐Bisection Task. Developmental Science 28, no. 5: e70060. 10.1111/desc.70060. 40781965 PMC12335016

[desc70193-bib-0017] Filardi, N. 2019. Reading Ability and Neural Configurations for Verbal and Nonverbal Information Processing, Master's thesis, Macquarie University.

[desc70193-bib-0018] Flöel, A. , S. Knecht , H. Lohmann , et al. 2001. “Language and Spatial Attention Can Lateralize to the Same Hemisphere in Healthy Humans.” Neurology 57, no. 6: 1018–1024. 10.1212/wnl.57.6.1018. 11571327

[desc70193-bib-0019] Frank, M. C. , M. Braginsky , D. Yurovsky , and V. A. Marchman . 2017. “Wordbank: An Open Repository for Developmental Vocabulary Data.” Journal of Child Language 44, no. 3: 677–694. 10.1017/S0305000916000209. 27189114

[desc70193-bib-0020] Gambrell, L. B. , and P. B. Jawitz . 1993. “Mental Imagery, Text Illustrations, and Children's Story Comprehension and Recall.” Reading Research Quarterly 28, no. 3: 265–276. 10.2307/747998.

[desc70193-bib-0021] Gerrits, R. , H. Verhelst , and G. Vingerhoets . 2020. “Mirrored Brain Organization: Statistical Anomaly or Reversal of Hemispheric Functional Segregation Bias?” Proceedings of the National Academy of Sciences 117, no. 25: 14057. 10.1073/pnas.2002981117. PMC7322015 32513702

[desc70193-bib-0022] Gignac, G. E. 2023. How2statsbook (Online Edition 2 ed.). Author.

[desc70193-bib-0023] Groen, M. A. , A. J. O. Whitehouse , N. A. Badcock , and D. V. M. Bishop . 2012. “Does Cerebral Lateralization Develop? A Study Using Functional Transcranial Doppler Ultrasound Assessing Lateralization for Language Production and Visuospatial Memory.” Brain and Behavior 2, no. 3: 256–269. 10.1002/brb3.56. 22741100 PMC3381631

[desc70193-bib-0024] Häberling, I. S. , A. Steinemann , and M. C. Corballis . 2016. “Cerebral Asymmetry for Language: Comparing Production With Comprehension.” Neuropsychologia 80: 17–23. 10.1016/j.neuropsychologia.2015.11.002. 26548403

[desc70193-bib-0026] Hebart, M. N. , A. H. Dickter , A. Kidder , et al. 2019. “THINGS: A Database of 1,854 Object Concepts and More Than 26,000 Naturalistic Object Images.” PLoS ONE 14, no. 10: e0223792. 10.1371/journal.pone.0223792. 31613926 PMC6793944

[desc70193-bib-0027] Horowitz‐Kraus, T. , M. Grainger , M. DiFrancesco , J. Vannest , S. K. Holland , and C. A. Consortium . 2015. “Right is Not Always Wrong: DTI and fMRI Evidence for the Reliance of Reading Comprehension on Language‐comprehension Networks in the Right Hemisphere.” Brain Imaging and Behavior 9, no. 1: 19–31. 10.1007/s11682-014-9341-9. 25515348

[desc70193-bib-0028] Horowitz‐Kraus, T. , Y. Wang , E. Plante , and S. K. Holland . 2014. “Involvement of the Right Hemisphere in Reading Comprehension: A DTI Study.” Brain Research 1582: 34–44. 10.1016/j.brainres.2014.05.034. 24909792 PMC4164572

[desc70193-bib-0029] Illingworth, S. , and D. V. M. Bishop . 2009. “Atypical Cerebral Lateralisation in Adults With Compensated Developmental Dyslexia Demonstrated Using Functional Transcranial Doppler Ultrasound.” Brain and Language 111, no. 1: 61–65. 10.1016/j.bandl.2009.05.002. 19525003 PMC2977530

[desc70193-bib-0030] Joffe, V. L. , K. Cain , and N. Marić . 2007. “Comprehension Problems in Children With Specific Language Impairment: Does Mental Imagery Training Help?” International Journal of Language & Communication Disorders 42, no. 6: 648–664. 10.1080/13682820601084402. 17852537

[desc70193-bib-0031] Johns, C. L. , K. M. Tooley , and M. J. Traxler . 2008. “Discourse Impairments Following Right Hemisphere Brain Damage: A Critical Review.” Lang Linguist Compass 2, no. 6: 1038–1062. 10.1111/j.1749-818X.2008.00094.x. 26085839 PMC4467466

[desc70193-bib-0032] Just, M. A. , S. D. Newman , T. A. Keller , A. McEleney , and P. A. Carpenter . 2004. “Imagery in Sentence Comprehension: An fMRI Study.” Neuroimage 21, no. 1: 112–124. 10.1016/j.neuroimage.2003.08.042. 14741648

[desc70193-bib-0033] Kohler, M. , H. A. D. Keage , R. Spooner , et al. 2015. “Variability in Lateralised Blood Flow Response to Language is Associated With Language Development in Children Aged 1–5 Years.” Brain and Language 145–146: 34–41. 10.1016/j.bandl.2015.04.004. 25950747

[desc70193-bib-0034] Kubota, E. C. , S. J. Joo , E. Huber , and J. D. Yeatman . 2019. “Word Selectivity in High‐level Visual Cortex and Reading Skill.” Developmental Cognitive Neuroscience 36: 100593. 10.1016/j.dcn.2018.09.003. 30318344 PMC6969272

[desc70193-bib-0035] Larsen, L. , S. Kohnen , L. Nickels , and G. McArthur . 2015. “The Letter‐Sound Test (LeST): A Reliable and Valid Comprehensive Measure of Grapheme–Phoneme Knowledge.” Australian Journal of Learning Difficulties 20, no. 2: 129–142. 10.1080/19404158.2015.1037323.

[desc70193-bib-0036] Levy, J. 1969. “Possible Basis for the Evolution of Lateral Specialization of the Human Brain.” Nature 224, no. 5219: 614–615. 10.1038/224614a0. 5346604

[desc70193-bib-0037] Lidzba, K. , K. Ebner , T.‐K. Hauser , and M. Wilke . 2013. “Complex Visual Search in Children and Adolescents: Effects of Age and Performance on fMRI Activation.” PLoS ONE 8, no. 12: e85168. 10.1371/journal.pone.0085168. 24376871 PMC3871624

[desc70193-bib-0038] Lidzba, K. , M. Staudt , M. Wilke , W. Grodd , and I. Krägeloh‐Mann . 2006. “Lesion‐Induced Right‐Hemispheric Language and Organization of Nonverbal Functions.” Neuroreport 17, no. 9: 929–933. 10.1097/01.wnr.0000221841.12632.d6. 16738490

[desc70193-bib-0039] Lindell, A. K. , and K. Hudry . 2013. “Atypicalities in Cortical Structure, Handedness, and Functional Lateralization for Language in Autism Spectrum Disorders.” Neuropsychology review 23, no. 3: 257–270. 10.1007/s11065-013-9234-5. 23649809

[desc70193-bib-0040] Liu, J. , A. Spagna , and P. Bartolomeo . 2022. “Hemispheric Asymmetries in Visual Mental Imagery.” Brain Structure and Function 227, no. 2: 697–708. 10.1007/s00429-021-02277-w. 33885966

[desc70193-bib-0041] Mellet, E. , L. Zago , G. Jobard , et al. 2014. “Weak Language Lateralization Affects Both Verbal and Spatial Skills: An fMRI Study in 297 Subjects.” Neuropsychologia 65: 56–62. 10.1016/j.neuropsychologia.2014.10.010. 25455569

[desc70193-bib-0042] Olulade, O. A. , A. Seydell‐Greenwald , C. E. Chambers , et al. 2020. “The Neural Basis of Language Development: Changes in Lateralization Over Age.” Proceedings of the National Academy of Sciences 117, no. 38: 23477–23483. 10.1073/pnas.1905590117. PMC7519388 32900940

[desc70193-bib-0043] Papadopoulou, A.‐K. , F. Vlachos , and M. Papadatou‐Pastou . 2022. “Cerebral Lateralization of Language in Children at Risk for Dyslexia: A Review of Neuroscientific Evidence.” Dialogues in Clinical Neuroscience & Mental Health 5, no. 2: 89–97. 10.26386/obrela.v5i2.228.

[desc70193-bib-0044] Pearson, J. 2019. “The Human Imagination: The Cognitive Neuroscience of Visual Mental Imagery.” Nature Reviews Neuroscience 20, no. 10: 624–634. 10.1038/s41583-019-0202-9. 31384033

[desc70193-bib-0045] Peirce, J. , J. R. Gray , S. Simpson , et al. 2019. “PsychoPy2: Experiments in Behavior Made Easy.” Behavior Research Methods 51, no. 1: 195–203. 10.3758/s13428-018-01193-y. 30734206 PMC6420413

[desc70193-bib-0046] Petit, S. , N. A. Badcock , and A. Woolgar . 2020. “Finding Hidden Treasures: A Child‐Friendly Neural Test of Task‐following in Individuals Using Functional Transcranial Doppler Ultrasound.” Neuropsychologia 146: 107515. 10.1016/j.neuropsychologia.2020.107515. 32504634 PMC7116264

[desc70193-bib-0047] Potdevin, D. , P. Adibpour , C. Garric , et al. 2023. “Brain Lateralization for Language, Vocabulary Development and Handedness at 18 Months.” Symmetry 15, no. 5: 989.

[desc70193-bib-0048] Powell, J. L. , G. J. Kemp , and M. García‐Finaña . 2012. “Association Between Language and Spatial Laterality and Cognitive Ability: An fMRI Study.” Neuroimage 59, no. 2: 1818–1829. 10.1016/j.neuroimage.2011.08.040. 21889594

[desc70193-bib-0049] Quin‐Conroy, J. E. , D. M. Bayliss , S. J. Bovell , et al. 2026. “Patterns of Language and Visuospatial Lateralisation in Three‐Year‐Old Children.” Neuropsychologia 226: 109434. 10.1016/j.neuropsychologia.2026.109434. 41850594

[desc70193-bib-0050] Quin‐Conroy, J. E. , D. M. Bayliss , S. G. Daniell , and N. A. Badcock . 2023. “Patterns of Language and Visuospatial Functional Lateralization and Cognitive Ability: A Systematic Review.” Laterality 29, no. 1: 63–96. 10.1080/1357650x.2023.2263199. 37771079

[desc70193-bib-0051] Quin‐Conroy, J. E. , Y. Chen , D. M. Bayliss , and N. A. Badcock . 2022. “Magic Hats and Teddy Bear Picnics: Language and Visuospatial Lateralisation Tasks for Children.” Laterality 27, no. 2: 232–256. 10.1080/1357650X.2021.2020808. 35019807

[desc70193-bib-0052] Quin‐Conroy, J. E. , P. A. Thompson , D. M. Bayliss , and N. A. Badcock . 2024. “Generalized Models for Estimating Cerebral Lateralisation of Young Children Using Functional Transcranial Doppler Ultrasound.” Human Brain Mapping 45, no. 13: e70012. 10.1002/hbm.70012. 39230061 PMC11372819

[desc70193-bib-0053] Rosopa, P. J. , M. M. Schaffer , and A. N. Schroeder . 2013. “Managing Heteroscedasticity in General Linear Models.” Psychological Methods 18, no. 3: 335–351. 10.1037/a0032553. 24015776

[desc70193-bib-0054] Salillas, E. , S. Benavides‐Varela , and C. Semenza . 2023. “The Brain Lateralization and Development of Math Functions: Progress Since Sperry.” Frontiers in Human Neuroscience 17: 1288154. 10.3389/fnhum.2023.1288154. 37964804 PMC10641455

[desc70193-bib-0055] Satz, P. , E. Strauss , M. Hunter , and J. Wada . 1994. “Re‐Examination of the Crowding Hypothesis: Effects of Age of Onset.” Neuropsychology 8, no. 2: 255–262. 10.1037/0894-4105.8.2.255.

[desc70193-bib-0056] Speed, L. J. , L. S. Eekhof , and M. Mak . 2024. “The Role of Visual Imagery in Story Reading: Evidence From Aphantasia.” Consciousness and Cognition 118: 103645. 10.1016/j.concog.2024.103645. 38241954

[desc70193-bib-0057] Stellern, J. , J. Collins , and M. Bayne . 1987. “A Dual‐Task Investigation of Language‐Spatial Lateralization.” Journal of Learning Disabilities 20, no. 9: 551–556. 10.1177/002221948702000907. 3694047

[desc70193-bib-0058] Stellern, J. , J. Collins , A. Cossairt , and B. Gutierrez . 1986. “Interference Asymmetry Involving Concurrent Tasks Performed by Native American Students.” Developmental Neuropsychology 2, no. 3: 241–255. 10.1080/87565648609540344.

[desc70193-bib-0059] Strauss, E. , P. Satz , and J. Wada . 1990. “An Examination of the Crowding Hypothesis in Epileptic Patients Who Have Undergone the Carotid Amytal Test.” Neuropsychologia 28, no. 11: 1221–1227. 10.1016/0028-3932(90)90057-u. 2290496

[desc70193-bib-0060] Szaflarski, J. P. , V. J. Schmithorst , M. Altaye , et al. 2006. “A Longitudinal Functional Magnetic Resonance Imaging Study of Language Development in Children 5 to 11 Years Old.” Annals of Neurology 59, no. 5: 796–807. 10.1002/ana.20817. 16498622 PMC2265796

[desc70193-bib-0061] Thompson, P. A. , K. E. Watkins , Z. V. J. Woodhead , and D. V. M. Bishop . 2023. “Generalized Models for Quantifying Laterality Using Functional Transcranial Doppler Ultrasound.” Human Brain Mapping 44, no. 1: 35–48. 10.1002/hbm.26138. 36377321 PMC9783456

[desc70193-bib-0062] Torgesen, J. , R. Wagner , and C. Rashotte . 1999. Test of word reading efficiency (TOWRE). Pro‐Ed.

[desc70193-bib-0063] Van der Haegen, L. , Q. Cai , and M. Brysbaert . 2012. “Colateralization of Broca's Area and the Visual Word Form Area in Left‐Handers: fMRI Evidence.” Brain and Language 122, no. 3: 171–178. 10.1016/j.bandl.2011.11.004. 22196742

[desc70193-bib-0064] Vigneau, M. , V. Beaucousin , P.‐Y. Hervé , et al. 2011. “What Is Right‐Hemisphere Contribution to Phonological, Lexico‐semantic, and Sentence Processing?: Insights From a Meta‐Analysis.” Neuroimage 54, no. 1: 577–593. 10.1016/j.neuroimage.2010.07.036. 20656040

[desc70193-bib-0065] Vingerhoets, G. 2019. “Phenotypes in Hemispheric Functional Segregation? Perspectives and Challenges.” Physics of Life Reviews 30: 1–18. 10.1016/j.plrev.2019.06.002. 31230893

[desc70193-bib-0066] Walenski, M. , E. Europa , D. Caplan , and C. K. Thompson . 2019. “Neural Networks for Sentence Comprehension and Production: An ALE‐Based Meta‐Analysis of Neuroimaging Studies.” Human Brain Mapping 40, no. 8: 2275–2304. 10.1002/hbm.24523. 30689268 PMC6597252

[desc70193-bib-0067] Wechsler, D. 1997. Wechsler Adult Intelligence Scale. In (3rd ed.). The Psychological Corporation.

[desc70193-bib-0068] Wechsler, D. 2014. Wechsler Preschool & Primary Scale of Intelligence—Australian and New Zealand Standardised Edition. Pearson Clinical. Retrieved 13/09 from https://www.pearsonclinical.com.au/products/view/539.

[desc70193-bib-0069] Wechsler, D. 2016. Wechsler Individual Achievement Test—Australian and New Zealand Standardised. Pearson Clinical. Retrieved 13/09 from https://www.pearsonclinical.com.au/products/view/588.

[desc70193-bib-0070] Weiss‐Croft, L. J. , and T. Baldeweg . 2015. “Maturation of Language Networks in Children: A Systematic Review of 22 Years of Functional MRI.” Neuroimage 123: 269–281. 10.1016/j.neuroimage.2015.07.046. 26213350

[desc70193-bib-0071] Wilson, A. C. , and D. V. M. Bishop . 2018. “Resounding Failure to Replicate Links Between Developmental Language Disorder and Cerebral Lateralisation.” PeerJ 6: e4217. 10.7717/peerj.4217. 29333343 PMC5764032

[desc70193-bib-0072] Zago, L. , L. Petit , E. Mellet , et al. 2016. “The Association Between Hemispheric Specialization for Language Production and for Spatial Attention Depends on Left‐hand Preference Strength.” Neuropsychologia 93: 394–406. 10.1016/j.neuropsychologia.2015.11.018. 26626612

